# Sialic Acid Metabolism: A Key Player in Breast Cancer Metastasis Revealed by Metabolomics

**DOI:** 10.3389/fonc.2018.00174

**Published:** 2018-05-28

**Authors:** Shao Thing Teoh, Martin P. Ogrodzinski, Christina Ross, Kent W. Hunter, Sophia Y. Lunt

**Affiliations:** ^1^Department of Biochemistry and Molecular Biology, Michigan State University, East Lansing, MI, United States; ^2^Department of Physiology, Michigan State University, East Lansing, MI, United States; ^3^Laboratory of Cancer Biology and Genetics, Center for Cancer Research, National Cancer Institute, National Institutes of Health, Bethesda, MD, United States; ^4^Department of Chemical Engineering and Materials Science, Michigan State University, East Lansing, MI, United States

**Keywords:** breast cancer metastasis, metabolomics, sialic acid, mass spectrometry, CMAS, PyMT, 4T1, 6DT1

## Abstract

Metastatic breast cancer is currently incurable. It has recently emerged that different metabolic pathways support metastatic breast cancer. To further uncover metabolic pathways enabling breast cancer metastasis, we investigated metabolic differences in mouse tumors of differing metastatic propensities using mass spectrometry-based metabolomics. We found that sialic acid metabolism is upregulated in highly metastatic breast tumors. Knocking out a key gene in sialic acid metabolism, *Cmas*, inhibits synthesis of the activated form of sialic acid, cytidine monophosphate-sialic acid and decreases the formation of lung metastases *in vivo*. Thus, the sialic acid pathway may be a new target against metastatic breast cancer.

## Introduction

Breast cancer is the most common cancer affecting women worldwide, with >1.6 million cases diagnosed each year. It is also one of the deadliest, causing >0.5 million deaths per year (1). The vast majority of breast cancer-related deaths occur due to metastasis, which is currently considered incurable (2). To develop and optimize treatment strategies for breast cancer, we must understand the molecular events that support metastasis.

The majority of studies on breast cancer metastasis have focused on identifying genetic or transcriptional changes associated with metastasis (3–5). More recently, the advent of wide-coverage, high-throughput metabolite profiling technologies such as liquid chromatography-mass spectrometry (LC-MS) have opened the possibility of investigating metabolic pathways that support metastasis. Dysregulation in energy metabolism is recognized as an emerging hallmark of cancer (6). However, metabolism is involved in far more than mere production of energy and biosynthetic intermediates: metabolites play central roles in cell signaling (7, 8), epigenetic regulation (9–11) and interaction with the microenvironment (12–15). Metastasis involves specific metabolic changes—for example, recent studies identified a requirement for proline in matrix-detached breast cancer cells (16), while breast cancer cells colonizing the lungs depend on pyruvate carboxylase for anaplerosis (17). In contrast, matrix-detached LKB1-deficient lung cancer cells require glutamine for survival (18). These add to a small but growing number of metabolic features specifically associated with metastasis as reviewed elsewhere (19–22). Hence, metabolomics—the comprehensive profiling of metabolites in biological systems—has the potential to shed new insights on the process of breast cancer metastasis.

Metastatic progression was originally thought to depend on clonal evolution—often accelerated by high mutation rates in cancer cells (23)—and the subsequent acquisition of prometastatic traits by a small subpopulation of cells within the primary tumor (24). However, this view has been challenged by experimental observations that genetic determinants of metastasis arise early in tumorigenesis (25, 26). Further, gene expression signatures within bulk primary tumors have been shown to predict their metastatic propensity (27, 28). This suggests that the majority of tumor cells within the primary tumor possess molecular features (e.g., gene expression, metabolite profiles) that reflect their metastatic ability (29). Thus, it should be possible to reveal metabolic pathways important for metastasis using metabolomics comparison of the primary tumors.

To identify metabolic pathways associated with breast cancer metastasis, we investigated metabolic differences between highly metastatic versus less-metastatic tumors using mass spectrometry-based metabolomics. We compared PyMT-driven tumors of differing metastatic propensities, generated by crossing MMTV-PyMT/FVB to mice of different backgrounds (30), and find increased levels of N-acetylneuraminic acid (Neu5Ac) in the highly metastatic tumors. Neu5Ac is the most common member of a family of structurally similar compounds termed “sialic acids”, and is also known simply as sialic acid. It is a nine-carbon monosaccharide commonly found as a terminal component of glycan chains on cell-surface glycoproteins and glycolipids. Cell surface sialylation mediates cell adhesion (31–34), immune recognition (35, 36) and signal transduction (37, 38) and has been previously implicated in cancer malignancy and metastatic potential (39–42). We therefore decided to investigate the role of sialic acid metabolism in breast cancer metastasis.

Following our initial analysis of highly metastatic versus less-metastatic breast tumors, we next compared sialic acid metabolism in highly metastatic 4T1 cancer cells versus non-metastatic 4T07 and 67NR cancer cells derived from the same BALB/c breast tumor (43). We find that metabolic flux to cytidine monophosphate (CMP)-sialic acid, the activated form of sialic acid, is increased in 4T1 cells, suggesting that upregulated sialic acid metabolism is a feature of highly metastatic breast cancer cells. We additionally find that genes in the sialic acid pathway, *GNE* and *CMAS*, are significantly associated with decreased breast cancer patient survival. Using CRISPR/Cas9, we then knocked out *Cmas* (the mouse homolog of *CMAS*) in 4T1 and 6TD1, which are metastatic mouse mammary tumor cell lines from different backgrounds, and find that orthotopically injected *Cmas*-KO cells have decreased metastatic capacity *in vivo*, confirming the importance of sialic acid metabolism in breast cancer metastasis. We show for the first time that targeting the sialic acid pathway in tumor cells reduces breast cancer metastasis.

## Materials and Methods

### PyMT Mouse Tumors

BL10 (MMTV-PyMT/FVB x C57BL/10J), BL6 (MMTV-PyMT/FVB x C57BL/6J), CAST (MMTV-PyMT/FVB x CAST/EiJ) or MOLF (MMTV-PyMT/FVB x MOLF/EiJ) mice were generated as previously reported (30). All animal use was performed in accordance with institutional and federal guidelines. Mice were sacrificed at humane end point as determined by the institute veterinarian and the tumors were harvested and flash-frozen for metabolomics analysis.

### Cell Lines and Culture Conditions

Mouse mammary carcinoma cell lines 4T1, 4T07, 67NR and 6DT1 were cultured in Dulbecco’s Modified Eagle Medium (DMEM) supplemented with 10% fetal bovine serum, 1% penicillin and streptomycin (P/S) and 1% glutamine and maintained in 37°C with 5% CO_2_.

### Metabolomics

Metabolites were extracted from tumors using a modification of the Bligh and Dyer method (44). For tumor metabolomics, flash-frozen tumor chunks were immersed in a mixture of HPLC-grade methanol and water (5:3 ratio) kept at −20°C and homogenized on a Precellys Evolution tissue homogenizer (15 s × 1 cycle, 10,000 rpm). HPLC-grade chloroform was added (methanol:water:chloroform ratio to 5:3:5), the mixture vortexed for 10 min at 4°C, and centrifuged at maximum speed (16,000 × *g*) for 10 min at 4°C to achieve phase separation. The methanol-water phase containing polar metabolites was separated and dried under a stream of nitrogen gas. The dried metabolite samples were stored at −80°C. Protein left from the extraction was dissolved in 0.2 mM KOH overnight, then quantified using Pierce BCA Protein Assay Kit (Thermo Fisher).

For cell culture experiments, cells were seeded in six-well plates at 150,000 cells/well and cultured for 24 h. For stable isotope labeling, media was switched to ^13^C-glucose-containing DMEM and samples collected at *T* = 0 (unlabeled), 15, 30, 60, 120, and 240 min after starting the experiment. Cells were washed with room-temperature saline and quenched by addition of cold (−75°C) methanol, followed by methanol–water–chloroform extraction as described above.

Dried metabolite extracts were resuspended in HPLC-grade water containing 1 μM piperazine-N,N′-bis (PIPES) added as an internal standard. To normalize sample concentrations, samples were resuspended at volumes corresponding to their protein quantification values. For amino acid analysis, 20 μl of resuspended sample was added to 80 μl MeOH and derivatized with 10 μl triethylamine and 2 μl benzylchloroformate. Samples with and without benzylchloroformate derivatization were transferred to HPLC vials for analysis.

Liquid chromatography tandem mass spectrometry (LC-MS/MS) analysis was performed with ion-pairing reverse phase chromatography using an Ascentis Express column (C18, 5 cm × 2.1 mm, 2.7 µm, Sigma-Aldrich) for separation and a Waters Xevo TQ-S triple quadrupole mass spectrometer operated in negative mode as mass analyzer. The LC parameters were as follows: autosampler temperature, 5°C; injection volume, 5 µl; column temperature, 50°C and flow rate, 400 µl/min. The LC solvents were solvent A: 10 mM tributylamine and 15 mM acetic acid in 97:3 water:methanol (pH 4.95) and solvent B: methanol. Elution from the column was performed over 12 min with the following gradient: *t* = 0, 0% solvent B, flow rate 0.4 ml/min; *t* = 1, 0% solvent B, flow rate 0.4 ml/min; *t* = 2, 20% solvent B, flow rate 0.3 ml/min; *t* = 3, 20% solvent B, flow rate 0.25 ml/min; *t* = 5, 55% solvent B, flow rate 0.15 ml/min; *t* = 8, 95% solvent B, flow rate 0.15 ml/min; *t* = 9.5, 95% solvent B, flow rate 0.15 ml/min; *t* = 10, 0% solvent B, flow rate 0.4 ml/min; *t* = 12, 0% solvent B, flow rate 0.4 ml/min. Mass spectra were acquired using negative-mode electrospray ionization operating in multiple reaction monitoring (MRM) mode. The capillary voltage was 3,000 V, and cone voltage was 50 V. Nitrogen was used as cone gas and desolvation gas, with flow rates of 150 and 600 l/h, respectively. The source temperature was 150°C, and desolvation temperature was 500°C. Argon was used as collision gas at a manifold pressure of 4.3 × 10^−3^ mbar. Precursor and product ion *m*/*z*, collision energies and source cone potentials were optimized for each transition using Waters QuanOptimize software. The MRM transitions are as listed in Table 1 in Supplementary Material. Peak processing was performed in MAVEN (45) and data for each sample was normalized to PIPES intensity. Statistical tests were performed in R using the t.test() and p.adjust() functions for Student’s *t*-test and Benjamini-Hochberg false discovery rate analysis, respectively.

### Determination of ^13^C-Labeled Metabolite Fractions Representing Flux Through the Hexosamine-Sialic Acid Pathway

The biosynthetic routes of uridine diphosphate (UDP)-N-acetylglucosamine, sialic acid and CMP-sialic acid were first examined to identify various groups on these molecules that may be labeled as distinct units. The units of labeling were identified as follows: UDP-N-acetylglucosamine has a glucose-derived hexose ring (C6), an acetyl group donated by acetyl-CoA (C2) and a UDP group comprising a ribose ring (C5) and uracil base (C4); sialic acid has a glucose-derived hexose ring (C6), an acetyl group donated by acetyl-CoA (C2), and a phosphoenolpyruvate (PEP)-derived moiety (C3); CMP-sialic acid has a glucose-derived hexose ring (C6), an acetyl group donated by acetyl-CoA (C2), a PEP-derived moiety (C3), and a CMP group comprising a ribose ring (C5) and cytosine base (C4).

Next, the MS/MS fragmentation patterns of UDP-N-acetylglucosamine, sialic acid and CMP-sialic acid were determined from the precursor and product ion *m*/*z* information in their MRM transitions. The changes to precursor and/or product ion nominal mass in isotopologues incorporating one or more units of labeling were calculated and saved as new MRM transitions (Table 3 in Supplementary Material). These transitions were validated from fragmentation mass spectra generated by a Q-TOF instrument operated in collision energy ramping mode.

Since conversion of ^13^C-glucose into UDP-N-acetylglucosamine, sialic acid and CMP-sialic acid through the hexosamine-sialic acid pathway would result in ^13^C-labeling of the hexose ring structure in these metabolites, the isotopic ratios of isotopologue species containing ^13^C-labeled hexose ring (with or without labeling of the various other constituents) were summed. The rate of increase of this hexose-labeled fraction therefore represents metabolic flux through the hexosamine-sialic acid pathway.

### Generation of *Cmas* Knockout Cell Lines

A single-stranded guide RNA (sgRNA) sequence targeting exon 2 of mouse *Cmas* was designed using Benchling^1^: 5′-TTCTACAATGGCGTCTAGTG-3′. CRISPR/Cas9 genome editing was performed using the Alt-R^®^ CRISPR-Cas9 System (Integrated DNA Technologies) following the supplied protocol but scaled to 12-well format. Briefly, Alt-R CRISPR-Cas9 cRNA containing *Cmas* target sequence, ATTO 550 fluorophore-linked Alt-R CRISPR-Cas9 tracRNA and Alt-R S/p Cas9 Nuclease 3NLS were combined to form ribonucleoprotein (RNP) complexes targeting *Cmas*. RNPs were delivered to the cells by reverse transfection using Lipofectamine RNAiMax (Thermo Fisher). 24 h after transfection, cells were FACS-sorted based on ATTO-550 fluorescence. Sorted cell pools were grown for two passages, then resuspended to a concentration of 1 cell/200 μl and seeded on 96-well plates (200 μl/well). Surviving clones were grown to confluence then passaged to 24-well plates. Genomic DNA was extracted using DNeasy Blood and Tissue Kit (Qiagen) to check for successful integration of indels into the target region. PCR primers were designed to amplify a region spanning exon 2 and exon 3 including the sgRNA target site as follows: forward 5′-GTTCAGGCACATACCACGATGC-3′, reverse 5′-GAAGAGTGGGGGATGAAGTGCT-3′. PCR products were agarose gel-purified, sequenced by ACGT Inc.^2^ and the results analyzed using Tracking of Indels by Decomposition^3^ (46) to identify the indels. Clones showing successful knockout were expanded and protein was harvested by RIPA buffer lysis. Protein samples were quantified with Pierce BCA Protein Assay Kit (Thermo Scientific) and diluted to uniform concentration (1 µg/µL). Western blotting was used to verify lack of Cmas protein expression in the knockout clones relative to wild-type (WT) cells or clones exhibiting WT sequence. To detect Cmas protein expression, rabbit polyclonal anti-Cmas HPA039905 (Sigma-Aldrich) and horseradish peroxidase-linked goat anti-rabbit IgG (heavy and light chain) antibody (Cell Signaling) were used for primary and secondary staining, respectively.

### Orthotopic Cell Line Injections

100,000 4T1 or 6DT1 cells were inoculated into the fourth mammary fat pad of 11–14-week-old BALB/c or 14-week old FVB/NJ female mice, respectively. Tumor growth was monitored, and end point was determined as tumor size reaching 2 cm or mice exhibiting excessive morbidity. Mice were euthanized 33–37 days (BALB/c) or 31 days (FVB) postinjection by carbon dioxide asphyxiation and cervical dislocation. For the second 6DT1 orthotopic injection experiment, FVB mice were euthanized at 24 days post-injection. Lungs were harvested, fixed in formalin, embedded in paraffin, sectioned and stained with hematoxylin and eosin (H&E) for histology analysis.

### Generation of Kaplan–Meier Survival Curves

Survival curves were generated with KM Plotter for Breast Cancer^4^ (47) using mRNA gene chip probes 205042_at and 218111_s_at for *GNE* and *CMAS*, respectively. Patients were split by median expression and the censor at threshold option was utilized. Redundant samples were removed and biased arrays excluded for the quality control.

### Analysis of Histology Images and Quantification of Lung Metastases

Microscope images of lung histology slides were analyzed using the “Fiji” distribution of ImageJ.^5^ Colors were converted using “Dichromacy > Tritanope” filter then split into three channels, and the “blue” channel showing high overall intensity was subtracted from the “red” channel showing high selective intensity in high-hematoxylin tumor tissue regions. Resultant images showing selective highlighting of tumor tissue region was then smoothed to reduce thresholding artifacts and finally thresholding applied to quantitate area of tumor tissue regions.

Student′s *t*-test (two-tailed, unpaired heteroscedastic) was applied to test for statistical significance of difference between average areas of each sample group.

For counting of discrete metastases, six sections were taken from the same lung at intervals of 200 µm, and unique metastatic lesions were counted over the sections and totaled. Only unique lesions were counted, i.e., a lesion already counted on one section would be excluded even if it is large enough to appear in the subsequent section. Chi-squared and Grubbs′ tests for outliers were performed with R statistical analysis software using the package “outliers.”

## Results

### Sialic Acid Levels Are Elevated in Highly Metastatic PyMT Tumors

As an initial investigation into the metabolic features of highly metastatic mammary tumors, we conducted metabolomics comparison of mammary tumors with differing metastatic propensities from novel mouse models based on the MMTV-PyMT model. These mice were generated by crossing male MMTV-PyMT/FVB mice to females of either C57BL/6J, C57BL/10J, MOLF/EiJ or CAST/EiJ background. The resulting F1 progeny, designated as BL6, BL10, MOLF, or CAST, respectively, consisted of two phylogenetically closely related pairs: {BL6, BL10} and {MOLF, CAST}. Mice of all four backgrounds develop PyMT-driven mammary tumors but display differing metastatic propensities, with the BL10 tumors being more metastatic than BL6 tumors, and the CAST tumors being more metastatic than the MOLF tumors (30). The mice were sacrificed at humane end point, primary tumors were collected, and polar metabolites were extracted from the tumor tissue and analyzed using a LC-MS/MS approach. The metabolite quantification results are provided in Data Sheet 1 in Supplementary Material.

To facilitate the identification of metabolites consistently correlated with metastasis, we first scaled the data to the average of low-metastatic tumors for each matched pair separately (i.e., data for the {BL6, BL10} pair were scaled to BL6 average, and data for the {MOLF, CAST} pair were scaled to MOLF average). Next, we combined the high-metastatic tumors from each matched pair (BL10 and CAST) into a “high-metastatic” group, and similarly put the low-metastatic tumors (BL6 and MOLF) into a “low-metastatic” group. We then ranked metabolites in increasing order of *p*-values (calculated using Student’s *t*-test) to identify the most significantly different metabolites between the high- and low-metastatic groups (Table 2 in Supplementary Material).

**Figure 1:**
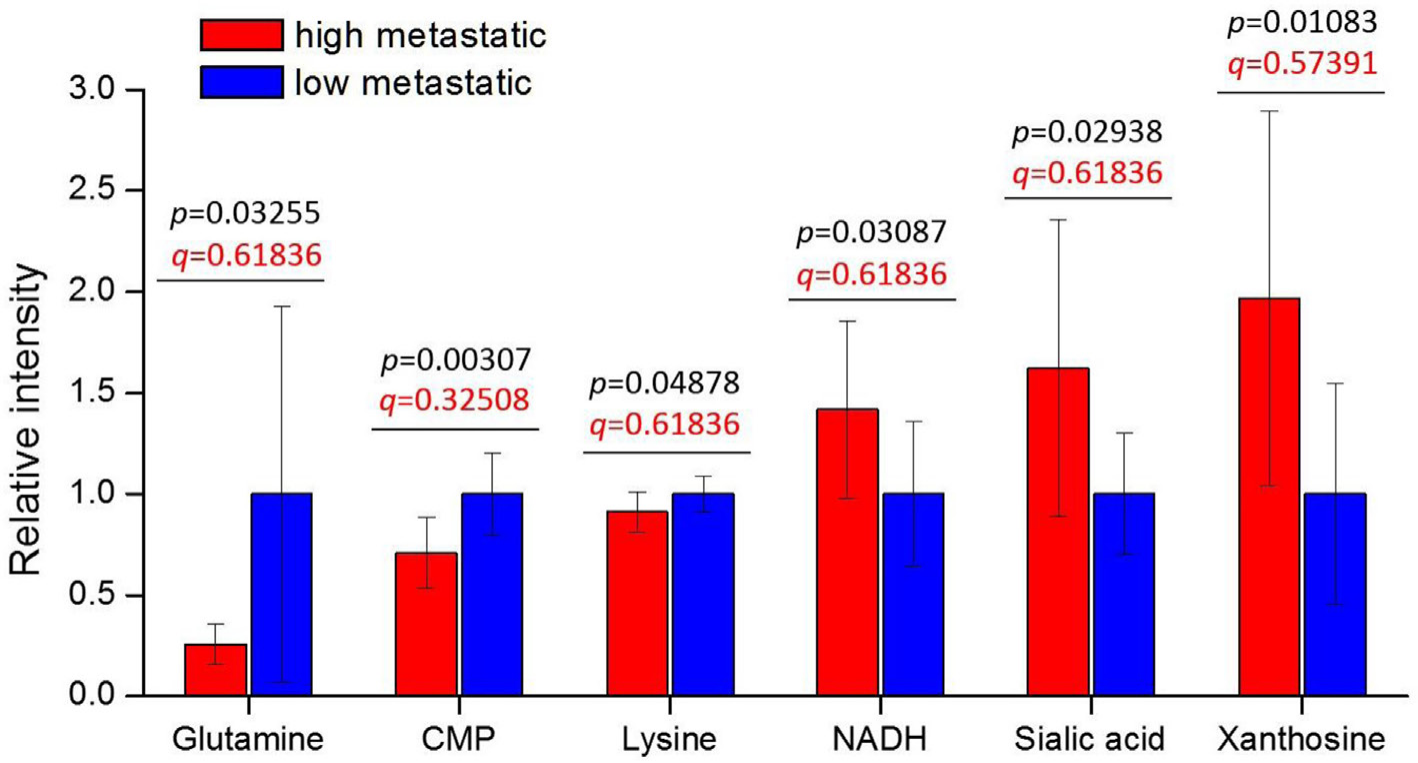
Tumor metabolite levels are significantly different between high- and low-metastatic groups. The highly metastatic group comprises tumors from BL10 and CAST mice, while low-metastatic group comprises tumors from BL6 and MOLF mice. Prior to sorting into high- and low-metastatic groups, each matched pair was separately scaled to the average of low-metastatic samples: {BL10, BL6} scaled to the BL6 average, {CAST, MOLF} scaled to the MOLF average. Metabolite levels are represented relative to low-metastatic group average. Values are the average of 10 individual mouse tumors (5 of each background, 2 backgrounds per group). Error bars represent SD. Student’s *t*-test *p*-values are shown in black, and false discovery rate *q*-values are shown in red.

We find six metabolites: CMP, xanthosine, sialic acid, NADH, glutamine, and lysine—with *p*-values < 0.05 (Figure 1). These metabolites are consistently increased or decreased in highly metastatic PyMT tumors relative to less metastatic tumors, across two different phylogenetically matched pairs, and therefore represent metabolites potentially associated with metastatic capability. Although the *q*-values calculated from false discovery rate analysis indicate a somewhat high probability of false positives (Table 2 in Supplementary Material), a number of these results are consistent with previous studies of metabolism in metastatic cells: higher NADH in the high-metastatic group may reflect increased TCA cycle activity, which is often identified as a feature of metastatic cells (48, 49); conversely, lower glutamine in the high-metastatic group may reflect the predisposition of metastasizing cells to utilize pyruvate, rather than glutamine, as a carbon source for TCA cycle anaplerosis (17). Since these aspects of metabolism have been previously reported, we instead focus on top-ranked metabolic features revealed by our analysis that have not been previously studied.

**Figure 2:**
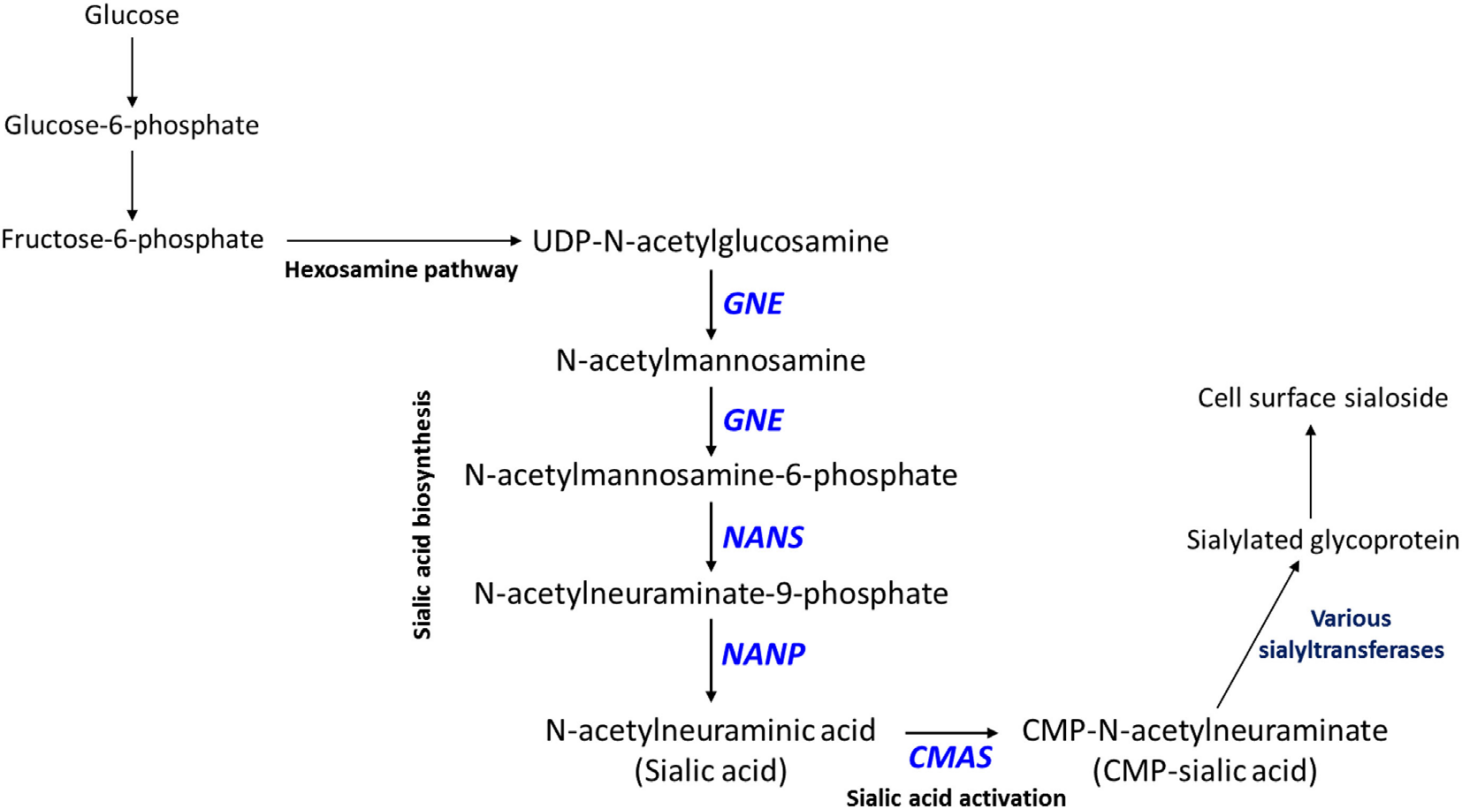
Sialic acid metabolic pathway. Sialic acid biosynthesis begins from uridine diphosphate (UDP)-N-acetylglucosamine, which is produced *via* the hexosamine pathway. *GNE* catalyzes the first committed step in sialic acid biosynthesis. Sialic acid is activated by coupling to cytidine monophosphate (CMP) *via* the action of *CMAS*, which produces CMP-sialic acid. CMP-sialic acid is used by sialyltransferases for sialylation of glycoproteins, which are then exported to the cell surface.

Interestingly, CMP (lower in high-metastatic group) and sialic acid (higher in the high-metastatic group) are relevant for the activation reaction of sialic acid, where upon a CMP group donated by cytidine triphosphate (CTP) is coupled to sialic acid to produce its activated form, CMP-sialic acid, which is subsequently used in cell-surface glycoprotein sialylation reactions (50). Hence, an increase in sialic acid may reflect increased flux through the sialic acid metabolic pathway (Figure 2), which could potentially lead to decreased levels of free CMP due to increased utilization of CTP for CMP-sialic acid synthesis. Increased or aberrant cell surface sialylation has been reported as a feature of cancer malignancy (39–41, 51). However, previous studies have focused on role of sialic acid on the cell surface (33, 34, 42, 52–54), and the importance of its metabolism remains unknown. This prompted us to further investigate the sialic acid metabolic pathway, which covers its biosynthesis as well as activation by coupling to CMP, as a potentially important metabolic feature in metastatic cancer cells.

### CMP-Sialic Acid Production Through the Sialic Acid Metabolic Pathway Is Increased in Metastatic 4T1 Cells

**Figure 3:**
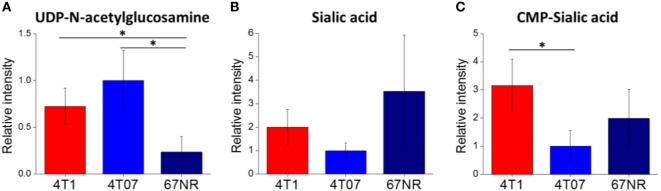
Cytidine monophosphate (CMP)-sialic acid is accumulated in highly metastatic 4T1 cells. 4T1, highly metastatic cell line; 4T07, less metastatic cell line; 67NR, non-metastatic cell line. All three cell lines were derived from the same BALB/c spontaneous mammary tumor. Metabolite abundances of **(A)** uridine diphosphate (UDP)-N-acetylglucosamine, **(B)** sialic acid, and **(C)** CMP-sialic acid are represented by peak intensities and are displayed relative to 4T07 averages for clarity. Values are the average of three biological replicates. Error bars represent SD. Statistically significant differences (*p*-value < 0.05) are marked with asterisks (*).

To confirm the importance of sialic acid metabolism in metastatic breast cancer, we investigated sialic acid pathway metabolites in another well-established model of metastasis: 4T1, 4T07, and 67NR cell lines. These are syngeneic cell lines, all derived from the same spontaneous mammary tumor, with greatly differing metastatic capabilities: while all three cell lines are tumorigenic, only 4T1 is highly metastatic, 4T07 is slightly metastatic and 67NR is non-metastatic (43, 55). These cell lines were derived from a different mouse background (BALB/c) and are not PyMT-driven; therefore, they provide additional validation of metastasis-associated metabolic features. The metabolite profiling results are provided in Data Sheet 2 in Supplementary Material. We find that the highly metastatic 4T1 cells indeed have the highest levels of CMP-sialic acid (the activated form of sialic acid), compared to the less metastatic cells (Figure 3). Slightly metastatic 4T07 cells accumulate higher levels of UDP-N-acetylglucosamine, the precursor for sialic acid biosynthesis, potentially indicating a decreased flux from this substrate to sialic acid (Figure 3). Although non-metastatic 67NR cells accumulate high levels of sialic acid, they have relatively low levels of the downstream metabolite CMP-sialic acid. These results suggest that CMP-sialic acid is efficiently produced in highly metastatic 4T1 cells, while sialic acid simply accumulates in non-metastatic 67NR cells, possibly due to lower conversion to CMP-sialic acid.

**Figure 4:**
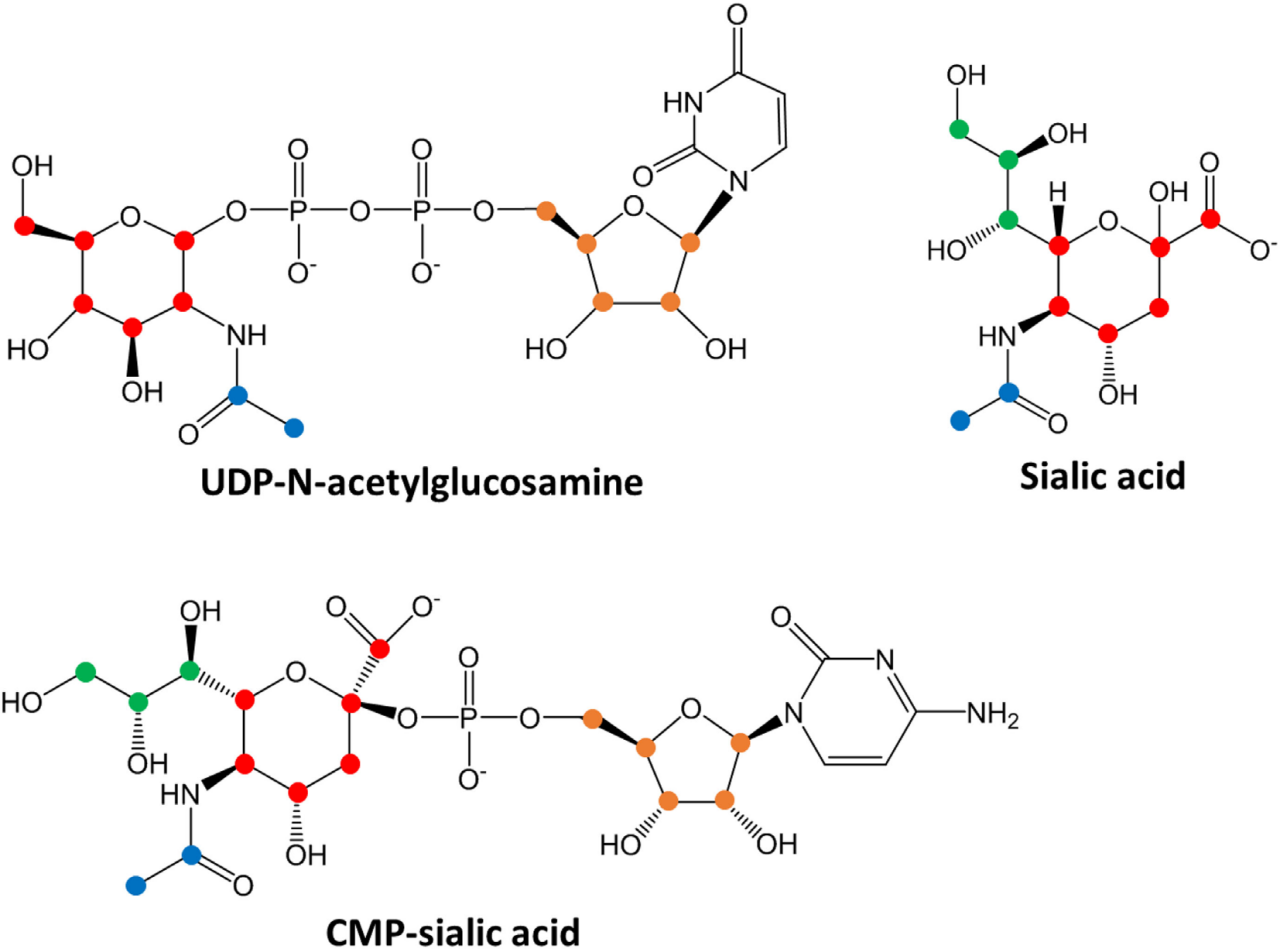
Chemical structures of sialic acid pathway intermediates. Carbon atoms derived from each biosynthetic component are denoted by different colors: red, glucose-derived hexose ring; green, PEP-derived moiety; blue, acetyl-CoA-derived moiety and orange, PRPP-derived ribose ring in the uridine monophosphate or cytosine monophosphate group. Biosynthesis of uridine diphosphate (UDP)-N-acetylglucosamine, sialic acid or cytidine monophosphate (CMP)-sialic acid from ^13^C-glucose would result in hexose ring carbons (red) becoming ^13^C-labeled, with additional labeling on the other colored carbons depending on extent of ^13^C-glucose incorporation into other metabolic pathways. By measuring the abundances of all isotopic species showing hexose ring (red) ^13^C-labeling, the amount of CMP-sialic acid production through the hexosamine-sialic acid pathway can be determined, and the rate of increase of this abundance reflects activity through the hexosamine-sialic acid pathway.

**Figure 5:**
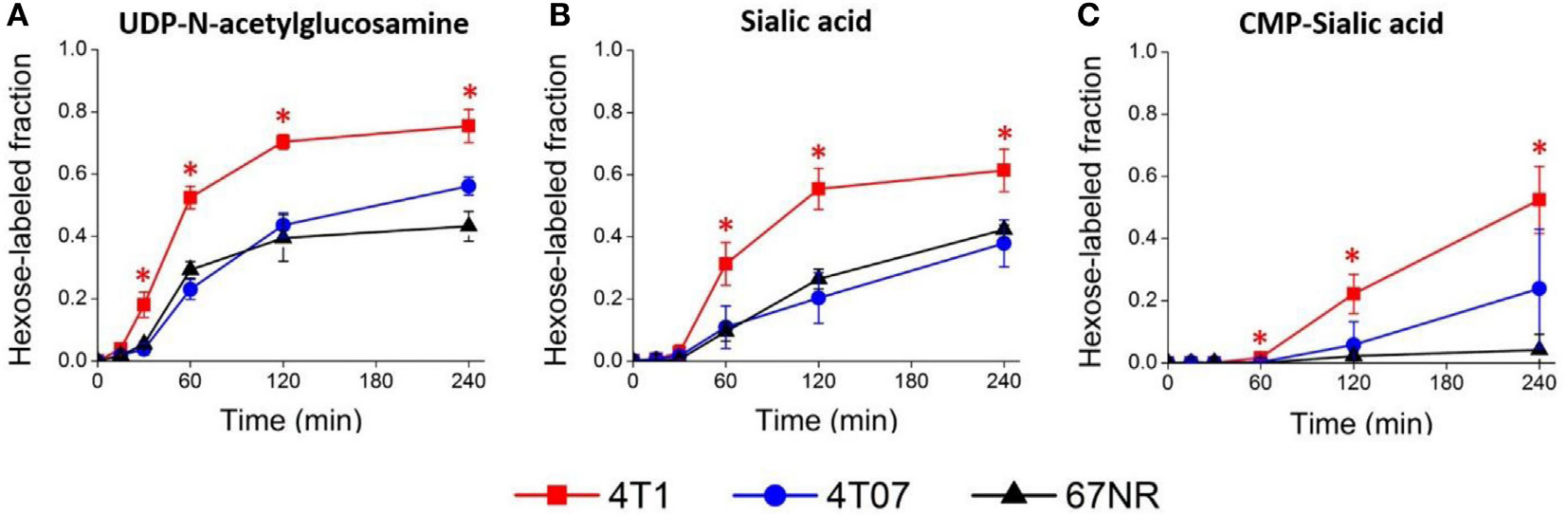
Sialic acid metabolism is upregulated in highly metastatic 4T1 cells. Highly metastatic 4T1 cells, slightly metastatic 4T07 cells, and non-metastatic 67NR cells were incubated with ^13^C-glucose media and extracted for metabolites at indicated time points. Hexose-labeled fractions of **(A)** uridine diphosphate (UDP)-N-acetylglucosamine, **(B)** sialic acid, and **(C)** cytidine monophosphate (CMP)-sialic acid reflect metabolic flux through the hexosamine-sialic acid pathway and were calculated by summing the isotopic ratios of species showing ^13^C-glucose labeled hexose rings. Values are the average of three biological replicates at each time point. Error bars represent SD. Time points showing statistically significant difference (*p*-value < 0.05) between 4T1 and the other two cell lines are marked with asterisks (*).

To verify that 4T1 cells have higher conversion of sialic acid to CMP-sialic acid compared to the less metastatic cell lines, we performed stable isotope labeling using ^13^C-glucose. We utilized a targeted profiling approach for specific ^13^C-labeled species of UDP-N-acetylglucosamine, sialic acid and CMP-sialic acid (see details in Material and Methods; results provided in Data Sheet 3 in Supplementary Material). Glucose flux through the hexosamine and sialic acid pathways would result in ^13^C-labeling of the hexose ring in UDP-N-acetylglucosamine, sialic acid and CMP-sialic acid (Figure 4). Hence, we examined the relative rate of hexose ring labeling for these metabolites. For each metabolite, this was calculated by summing the isotopic ratios of all species containing a ^13^C-labeled hexose ring (Table 3 in Supplementary Material). We find that 4T1 cells had the highest rate of hexose ring labeling of UDP-N-acetylglucosamine, sialic acid and CMP-sialic acid, with statistically significant (*p*-value < 0.05) differences in hexose-labeled fraction of each of these metabolites for the *T* = 60, 120 and 240 min time points (Figure 5). Together with the elevated level of CMP-sialic acid in 4T1 observed previously (Figure 3C), this result strongly supports our hypothesis that highly metastatic 4T1 cells have the highest glucose flux to CMP-sialic acid. Although 4T07 and 67NR showed similar kinetics for the UDP-N-acetylglucosamine and sialic acid hexose-labeled fractions (Figures 5A,B), the higher abundance of UDP-N-acetylglucosamine in 4T07 (Figure 5A) and sialic acid in 67NR (Figure 5B) indicate that the absolute labeling rate (in terms of molecules converted per unit time) is higher for UDP-N-acetylglucosamine in 4T07, and higher for sialic acid in 67NR. This difference is more appropriately visualized when the hexose-labeled fractions of Figure 5 are multiplied by the time zero unlabeled metabolite abundances shown in Figure 4 (Image 1 in Supplementary Material). The moderately high rate of UDP-N-acetylglucosamine labeling (Image 1A in Supplementary Material) and very low rate sialic acid labeling (Image 1B in Supplementary Material) suggests a metabolic bottleneck in the conversion of UDP-N-acetylglucosamine to sialic acid in 4T07. On the other hand, 67NR has surprisingly high absolute labeling of sialic acid (Image 1A in Supplementary Material) despite low labeling of its upstream metabolite, UDP-N-acetylglucosamine (Image 1A in Supplementary Material) possibly hinting at an alternative pathway of sialic acid production that bypass UDP-N-acetylglucosamine. Regardless, both 4T07 and 67NR display drastically reduced rate of CMP-sialic acid labeling compared to 4T1 (Image 1C in Supplementary Material), suggesting that production of CMP-sialic acid (i.e., the activated form, rather than sialic acid itself), is the key metabolic feature that supports metastasis.

### Sialic Acid Pathway Genes *GNE* and *CMAS* Are Associated With Decreased Patient Survival

**Figure 6:**
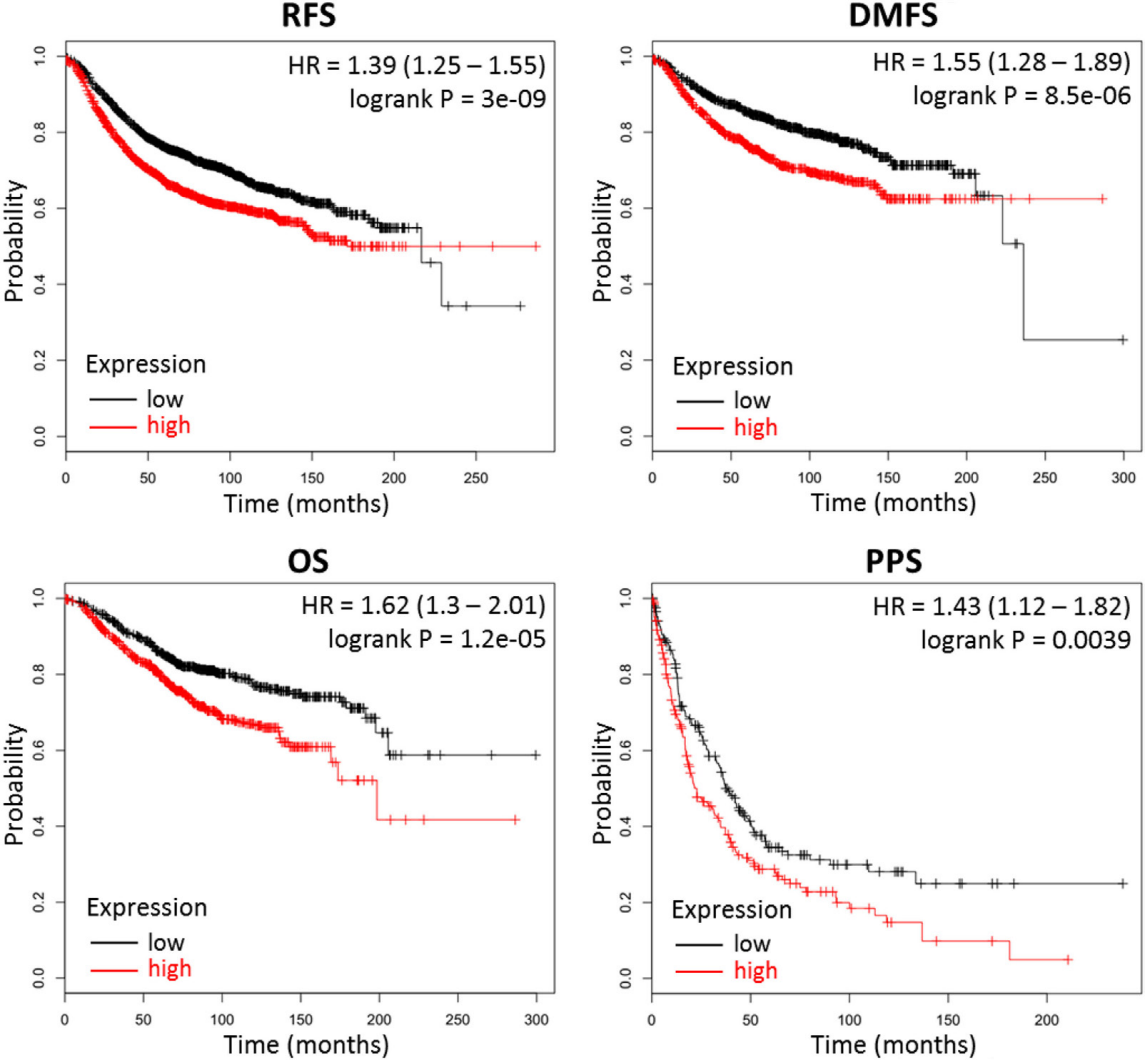
Combined *GNE* and *CMAS* expression is strongly associated with decreased survival in breast cancer patients. Kaplan–Meier survival curves were generated by KM Plotter (47) using transcription data and patient survival information extracted from Gene Expression Omnibus (GEO) datasets. Abbreviations: RFS, relapse-free survival; DMFS, distant metastasis-free survival; OS, overall survival; PPS, postprogression survival. Overall expression of *GNE* and *CMAS* is represented by the mean of individual gene expression values. The median expression value was used as the cut-off value for determining high- versus low-expression patient groups.

Our results above indicate that high production of CMP-sialic acid through the sialic acid pathway is a metabolic feature associated with highly metastatic breast tumor cells. We sought to verify the clinical significance of this pathway using breast cancer patient data. To investigate whether high expression of genes in the sialic acid pathway is associated with breast cancer patient survival, we used KM Plotter (47), which extracts patient survival data from publicly available Gene Expression Omnibus (GEO) datasets to generate Kaplan–Meier curves. Using median expression as the cut-off for defining high expression, we find that two key genes in the sialic acid pathway are strongly associated with decreased distant metastasis free survival: glucosamine (UDP-N-acetyl)-2-epimerase/N-acetylmannosamine kinase (*GNE*), which catalyzes the first committed step in sialic acid production from UDP-N-acetylglucosamine; and N-acylneuraminate cytidylyltransferase (*CMAS*), which catalyzes the final step of CMP-sialic acid production (Table 1). In addition, *GNE* is strongly associated with decreased postprogression survival while *CMAS* was significantly associated with relapse-free survival. Remarkably, the mean value of *GNE* and *CMAS* expression, which provides a better measure of overall pathway activity, is even more strongly associated with all four measures of survival: relapse-free survival, distant metastasis-free survival, overall survival and postprogression survival (Figure 6). These results highlight the importance of the sialic acid pathway in breast cancer metastasis.

**Table 1:** Summary of hazard ratios and *p*-values from Kaplan–Meier curves.

Survival measure	No. of usable cases	*GNE*		*CMAS*		*GNE* + *CMAS*	
		Hazard ratio	*p*-Value	Hazard ratio	*p*-Value	Hazard ratio	*p*-Value
RFS	3,951	1.04 (0.93–1.16)	0.5217	1.36 (1.22–1.52)	2.20E−08	1.39 (1.25–1.55)	3E−09
DMFS	1,746	1.36 (1.12–1.66)	0.0017	1.26 (1.04–1.53)	0.019	1.55 (1.28–1.89)	8.50E−06
OS	1,402	1.39 (1.12–1.73)	0.0028	1.31 (1.06–1.62)	0.014	1.62 (1.3–2.01)	1.20E−05
PPS	414	1.43 (1.12–1.82)	0.0042	1.21 (0.95–1.54)	0.13	1.43 (1.12–1.82)	0.0039

*Association of breast cancer patient survival with expression of *GNE, CMAS* or *GNE* and *CMAS* combined (mean expression) is represented by hazard ratio and *p*-value, with a higher hazard ratio and smaller *p*-value indicating stronger association with decreased survival. 95% confidence intervals of the hazard ratios are indicated in parentheses*.

*RFS, relapse-free survival; DMFS, distant metastasis-free survival; OS, overall survival; PPS, postprogression survival*.

### *Cmas* Knockout Decreases Breast Cancer Metastasis *In Vivo*

Our results so far indicate that sialic acid pathway activity is correlated with metastasis. To investigate whether the sialic acid metabolic pathway directly supports metastasis, we targeted a key pathway gene using CRISPR/Cas9. As mentioned above, CMP-sialic acid, rather than sialic acid itself, seems to be the key metabolite supporting metastasis, most likely through its role in cell surface sialylation. Hence, inhibition of CMP-sialic acid production could attenuate metastasis. Analysis of patient data indicated that both *GNE* and *CMAS*, which catalyze the first and last steps of CMP-sialic acid production, respectively, are strongly associated with decreased distant metastasis free survival. Since sialic acid may be produced *via* reactions that bypass *GNE*, such as through scavenging pathways, we focused on targeting *CMAS*.

**Figure 7:**
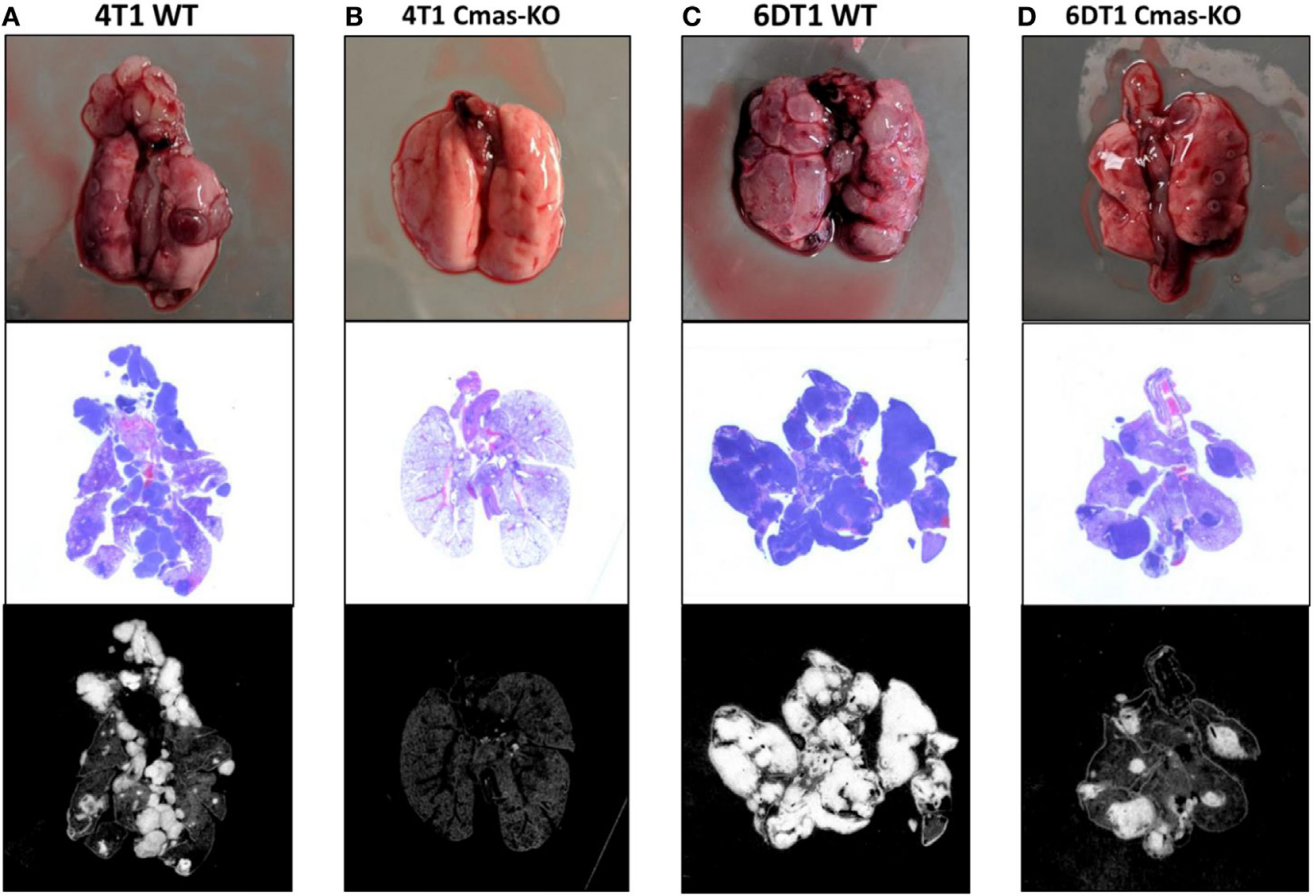
Representative images of lungs from mice injected with *Cmas* WT and KO cells. **(A)** Lungs from mice orthotopically injected with 4T1 wild-type cells showing high extent of metastasis. **(B)** Lungs from mice injected with 4T1 *Cmas*-KO cells showing almost no metastasis. **(C)** Lungs from mice injected with 6DT1 wild-type cells showing extremely high extent of metastasis. **(D)** Lungs from mice injected with 6DT1 *Cmas*-KO cells showing relatively low extent of metastasis. Top row: lungs photographed at necropsy. Middle row: histology images showing hematoxylin and eosin (H&E) staining. Metastatic tumor tissue is identified by nuclei-dense regions showing high hematoxylin (blue) staining. Bottom row: histology images processed in ImageJ to highlight the regions used for area quantification. Images are not perfectly matched in scale.

Using CRIPSR/Cas9, we generated *Cmas* (mouse ortholog of *CMAS*) knockouts of 4T1 cells. To avoid bias from using a single mouse background, we additionally generated *Cmas* knockouts of 6DT1, a highly metastatic breast cancer cell line generated from an MMTV-Myc driven tumor in the FVB mouse background (56). We isolated a number of clones by serial dilution, then confirmed knockout of *Cmas* at the gene and protein expression levels by sequencing and Western blotting, respectively (Images 2 and 3 in Supplementary Material). As an initial test, we performed orthotopic injections of WT and two confirmed *Cmas*-KO clones of 4T1 cells into the mammary fat pads of isogeneic BALB/c mice. All mice developed tumors that grew rapidly, indicating that *Cmas*-KO did not impair the ability of 4T1 cells to proliferate or form tumors *in vivo*. The mice were sacrificed at humane end point (33–37 days postinjection) and inspected for pulmonary metastasis. The mice injected with WT 4T1 cells display extensive pulmonary metastasis, with visible metastatic tissue growth on the lung surface as well as lung-adjacent regions including the sternum, ribcage, and diaphragm (Figure 7A). In contrast, mice injected with *Cmas*-KO 4T1 cells have hardly any visible surface metastases on the lungs (Figure 7B).

To validate these results and verify that this is not a 4T1 cell line-specific effect, we subsequently orthotopically injected WT and two confirmed *Cmas*-KO clones of 6DT1 cells into the mammary fat pads of isogeneic FVB mice. To control for potential effects of the Cas9 transfection and cell sorting process on the cells’ metastatic capability, we also injected an additional set of mice with a 6DT1 clone that had underwent the same transfection and cell sorting process as the *Cmas*-KO clones, but was found to have WT *Cmas* sequence and protein expression (Images 2 and 3 in Supplementary Material). All injected mice developed tumors that grew even more rapidly than the 4T1 tumors, and the mice were sacrificed at 31 days post-injection. As before, we observe extensive metastatic spread to the lungs in the mice injected with cells with WT *Cmas* (Figure 7C). Interestingly, lung metastasis is also observed in the *Cmas*-KO injected mice, although to a visibly lesser degree compared to the WT injected mice (Figure 7D). This may reflect: (i) a higher intrinsic aggressiveness of 6DT1 cells compared to 4T1 cells; (ii) a lower dependence on CMP-sialic acid for metastasis in 6DT1 cells; and/or (iii) lower intrinsic resistance of FVB mice toward metastasis, compared to BALB/c mice.

**Figure 8:**
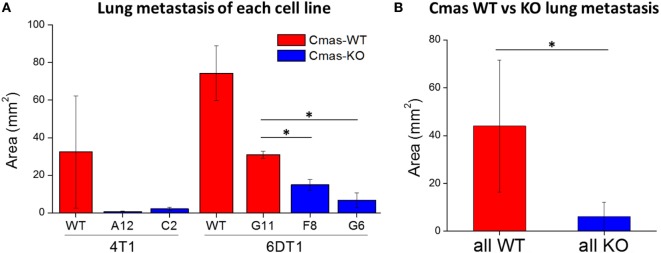
Orthotopic injection of *Cmas*-KO cells result in less lung metastasis compared to *Cmas*-wild-type (WT). **(A)** Average metastatic tissue area for each injected cell line. Values are the average of three (4T1 WT, 6DT1 G6) or two (all other cell lines) biological replicates, and error bars represent SD. **(B)** Average metastatic tissue area across all *Cmas* WT injections versus all *Cmas*-KO injections. Values are the average of seven total samples in the WT group and nine total samples in the KO group. Error bars represent SD. Statistically significant differences (*p*-value < 0.05) are marked with asterisks (*).

To quantify the extent of lung metastasis, we analyzed the total area of metastatic tissue from images of H&E-stained sections of paraffin-embedded lungs. Compared to the 6DT1 clone with WT Cmas (G11), both 6DT1 *Cmas*-KO clones show significantly smaller total metastatic area (Figure 8A). On average, 6DT1 WT cells show larger total metastatic area than the G11 WT clone; however, the difference is not statistically significant due to larger variation in the values for 6DT1 WT lung metastases (Figure 8A). Similarly, 4T1 *Cmas*-KO clones result in smaller metastatic area relative to 4T1 WT, although the difference is not statistically significant due to large variation in the WT samples. To compare the extent of metastasis across all *Cmas* WT versus all *Cmas*-KO samples overall, we assigned the 4T1 WT, 6DT1 WT and 6DT1 G11 (clone with WT sequence) samples into a “*Cmas*-WT” group, and likewise assigned samples corresponding to the 4T1 *Cmas*-KO clones (A12 and C2) and 6DT1 *Cmas*-KO clones (F8 and G6) into a “*Cmas*-KO” group. Between these two groups, we find a high statistical significance (*p-*value = 0.0106) in lung metastatic area (Figure 8B), indicating that knockout of *Cmas* decreased the metastatic potential of orthotopically injected cancer cells.

**Figure 9:**
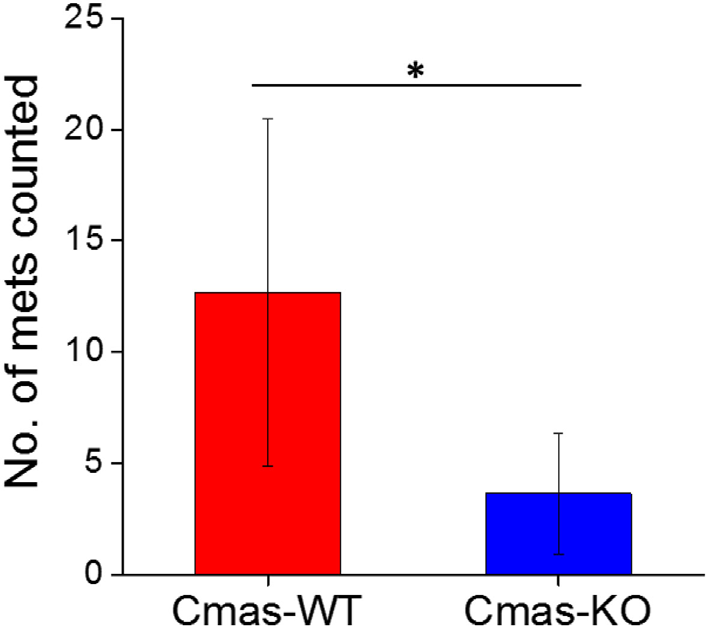
Orthotopically injected *Cmas*-KO cells form fewer discrete lung metastases compared to *Cmas*-wild type (WT). Each data point is the total number of discrete metastatic lesions counted over six sections, taken at 200 µm depth intervals, of the same lung. Values are the average of six (*Cmas*-WT) or eight (*Cmas*-KO) biological replicates, and error bars represent SD. The asterisk (*) denotes statistical significance of difference (*p*-value < 0.05).

Finally, to determine whether the decreased lung metastatic area in *Cmas*-KO injected mice was due to decreased number of lung metastases or slower growth of metastatic tissue, we again performed orthotopic injection of FVB mice with 6DT1 cells, and this time sacrificed the mice at an earlier time point of 24 (rather than 31) days post-injection. At this time point, the metastatic lesions are smaller and hence countable as discrete lesions. Two different *Cmas*-WT clones and three different *Cmas*-KO clones were used. To gain a more representative view of the distribution of metastatic lesions throughout the lungs, multiple histology sections of each paraffin-embedded lung were taken at depth intervals of 200 µm, H&E stained and the total number of discrete metastatic lesions counted over all sections from the same lung. One of the *Cmas*-KO samples was determined to be an outlier according to the chi-squared test (*p*-value = 0.01125) as well as the Grubbs’ test (*p*-value = 0.0003802) and was hence removed from the dataset (Image 4 in Supplementary Material). We observe a significant difference (*p*-value = 0.03544) between *Cmas*-WT and *Cmas*-KO in the number of discrete lung metastases formed (Figure 9), with *Cmas*-WT injected mice having 12.7 lung metastases on average (*n* = 6) and *Cmas*-KO injected mice having 3.6 lung metastases on average (*n* = 8). Hence, knockout of *Cmas*, a key gene in sialic acid metabolism, may affect metastatic potential by decreasing the rate of successful metastatic seeding.

## Discussion

In this study, we directly demonstrate elevated sialic acid metabolism in highly metastatic tumor cells and show the direct relevance of this pathway by successfully decreasing the metastatic potential of two different highly metastatic breast cancer cell lines *via* knockout of a key pathway gene. A correlation between the metastatic potential of cancer cells and sialylation of their cell surface was discovered as early as the 1980s by Yogeeswaran and Salk (42). However, until more recently, studies of sialic acid metabolism—including its production and subsequent incorporation into cell surface proteins and lipids—had been more limited, perhaps due to the difficulty of directly measuring metabolite levels prior to the advent of mass spectrometry-based metabolomics. It should be noted that there are other sialic acids besides Neu5Ac, albeit at much lower abundance in mammalian cells. One example is N-glycosylneuraminic acid (Neu5Gc), which is produced in its CMP-conjugated form *via* hydroxylation of CMP-Neu5Ac by the CMAH gene (57). The CMAH gene has been found to be mutated and nonfunctional in humans (58, 59), although Neu5Gc has been detected in human tissue, likely from dietary uptake (60, 61), and may be a potential oncogenic trigger (62, 63). Another is deaminoneuraminic acid, also known as ketodeoxynonulosonic acid (KDN) (64), which is synthesized through a similar biological pathway as Neu5Ac but using mannose rather than N-acetylmannosamine as substrate (65). Altered KDN levels have been implicated in ovarian, throat and prostate cancers (66–68). Consistent with the much lower abundance of these sialic acids (66, 69), our method did not detect either the free nor CMP-conjugated forms of Neu5Gc and KDN in our mouse tumor and cell samples.

Our findings constitute an addition to a small number of studies that have specifically explored the role of sialic acid metabolism in cancer cells. Previously, Kohnz et al. (37) found increased glycolytic carbon incorporation into the sialic acid pathway metabolites upon oncogenic transformation of MCF10A cells and also identified a number of glycoproteins whose levels were significantly affected by increased or decreased sialic acid pathway flux. These included important oncogenic signaling proteins such as epidermal growth factor receptor, as well as the breast cancer stem cell marker CD44. The authors also noted that *CMAS* knockdown of MDA-MB-231–derived cells impaired serum-free survival and tumor xenograft growth in immune-deficient mice. Interestingly, *CMAS* knockdown resulted in transcriptional downregulation of many genes, including those involved in immunological responses such as cytokines (IL-1 and IL-6) and a number of chemokine ligands and receptors. It seems likely that this widespread transcriptional reprogramming was, at least in part, due to key signaling pathways being disrupted by the loss of sialylation on cell surface proteins with signal transduction functions. A previous study found that increased sialic acid pathway flux in SW1990 pancreatic cancer cells selectively increased the abundance of certain sialylated glycoproteins while having a minimal effect on others (31); however, it was not clear whether the increase in these sialoglycoproteins was due to upregulation at the transcriptional level or increased sialylation rate of the glycoproteins. The results of Kohnz et al. (37) seem to suggest that at least some of these changes in sialoglycoproteins could be transcriptional in origin. Notably, neither of these studies specifically investigated the role of sialic acid pathway flux in supporting metastasis, although Almaraz et al. (31) noted that increased sialic acid pathway activity enhanced CD44-mediated adhesion to selectins as well as integrin-mediated cell mobility on collagen and fibronectin, which may have implications for the metastatic potential of the cells.

Our study establishes the importance of the sialic acid pathway using metabolomic approaches rather than gene expression approaches. Gene expression alone would not have indicated the importance of the sialic acid biosynthesis pathway in metastasis. RNA-seq data show that out of nine genes in the hexosamine/sialic acid pathway, only Gne has a meaningful difference (FDR = 0.6) between the high-metastatic (BL10, CAST) and low-metastatic (BL6, MOLF) groups (Image 5 in Supplementary Material). For the BALB/c-derived cell lines, microarray data from publicly available GEO Datasets show higher Gfpt1, Gfpt2, Pgm3, and Cmas expression in highly metastatic 4T1 cells, and higher Uap1, Gne, Nans, and Nanp expression in non-metastatic 67NR cells (Images 6–8 in Supplementary Material). This trend, while consistent across all three datasets, does not point to the upregulation of the hexosamine/sialic acid pathway at the gene expression level. Additionally, the less-metastatic 4T07 show highest expression out of the three lines for Gfpt1, Pgm3, and Cmas (Image 8 in Supplementary Material), rather than falling between 4T1 and 67NR as would be expected if pathway gene expression were perfectly correlated with metastatic capacity. In partial agreement with gene expression data for Cmas, Western blotting show markedly lower Cmas protein expression in 67NR cells, although the difference between 4T1 and less-metastatic 4T07 are not visibly quantifiable (Image 9 in Supplementary Material). In short, the differences in sialic acid metabolite levels or metabolic flux through the pathway are not reflected by differences at the gene expression level. This is not surprising given the fact that beyond transcriptional- or translational-level regulation, posttranslational modifications and metabolic control mechanisms (e.g., substrate/product ratio and negative feedback *via* inhibitory products) exert significant influence on enzymatic activities (11, 70). These observations indicate that the significance of this pathway would likely be missed solely by comparisons at the gene expression level and highlight the utility of metabolic studies (e.g., measuring metabolite levels and metabolic fluxes) as a powerful complement to traditional genome or gene expression-based comparative studies.

In our study, we establish a correlation between intracellular sialic acid metabolite pool sizes and metastatic capability of breast cancer cells. Although we did not quantify the extent of cell surface sialylation, it is reasonable to expect that increased metabolite levels and metabolic fluxes in the sialic acid pathway would result in increased sialylation of cell surface glycoproteins and glycolipids. Hsieh et al showed that fructose feeding of human pancreatic ductal adenocarcinoma (PDAC) cells resulted in upregulation of β-galactoside α2,6-sialyltransferase 1 (ST6Gal1) and increased levels of cell surface α2,6-sialylation, likely due to increase substrate availability as excess fructose is converted into hexosamine and sialic acid pathway metabolites (32). Notably, ST6Gal1 overexpressing cells showed increased invasiveness and metastatic potential when injected into immunodeficient mice, suggesting that α2,6-sialylation enhances metastasis. Other studies have also demonstrated the correlation between sialyltransferase activity, cell surface sialylation and metastatic capability of PDAC and breast cancer cells (71, 72). This points to sialyltransferases as another possible target for inhibiting metastasis through disrupting cell surface sialylation. Indeed, sialyltransferase inhibition by a rationally designed glycomimetic has been successfully employed to inhibit metastatic spread (73, 74).

It is not clear whether specific patterns of sialylation (e.g., α2,3- or α2,6-sialylation), or a general increase in cell surface sialylation, predominantly drives the phenotypic differences in cells with increased or decreased sialic acid pathway activity. It is likely dependent on the specific cancer cell type. Both α2,3-sialylation (33, 35, 53) and α2,6-sialylation (52, 75) have been implicated in acquisition of potential metastatic-related traits, such as invasion or immune evasion. Interestingly, Bassagañas et al. (53) reported that while α2,6-sialylation increased adhesion to extracellular matrix (ECM) proteins, it did not enhance the migratory phenotype of PDAC cells, while α2,3-sialylation had a much more pronounced effect on migration. Interestingly, the increase in one type of sialylation by overexpression of a sialyltransferase (ST3Gal3) may come at the expense of another type of sialylation, presumably due to decreased availability of the CMP-sialic acid substrate for other sialyltransferases. Additionally, α2,8-sialylation has also been correlated with proliferation, migration and invasion in breast cancer cells (34). Since specific classes of sialyltransferases catalyze each type of sialylation, it may be difficult to target the most relevant sialyltransferase(s) to inhibit cancer metastasis. Therefore, an upstream reaction such as *CMAS* may be a more promising target, since attenuation of this single gene through knockout or knockdown reduces the availability of CMP-sialic acid, the substrate for all sialylation reactions. Another possible target is the sialic acid transporter Slc35a1, which shuttles activated CMP-sialic acid from the nucleus to the Golgi apparatus, and whose knockdown can similarly impact cell surface sialylation by decreasing CMP-sialic acid availability to the sialyltransferases (35). However, it is not known at this point whether multiple transporter genes exist that may need to be individually targeted in different cell types.

The relationship between sialylation, cell adhesion and the phenotypes relating to cell mobility such as migration and invasion is not clear. In general, sialylation seems to increase adhesion to ECM proteins such as selectins or fibronectin, while also enhancing migration and invasion (31–34). However, Bassagañas et al. (53) reported that only α2,3-sialylation correlated with migratory ability, while α2,6-sialylation correlated with ECM adhesion but not migration. In contrast, Yuan et al. (52) reported that removal of α2,6-sialic acid residues in highly metastatic MDA-MB-231 cells by sialidase treatment *increased* adhesion of the cells to ECM proteins such as collagen IV and fibronectin, while having no significant effect on *in vitro* migration or invasion. Another study reported enhanced mobility of human keratinocyte HaCaT cells, concurrent with an upregulation in mesenchymal markers consistent with the epithelial–mesenchymal transition, upon inhibition of cell surface sialylation (75). Although most studies linked metastatic ability with the acquisition of migratory and invasive phenotypes, this is likely not the only role of cell surface sialylation in supporting metastasis. Cell surface sialylation also affects cancer cells’ interaction with the components of the immune system. Examples include: attenuating natural killer (NK) cell-mediated innate immune response by masking the cancer cells from immune surveillance (35, 36); directly inhibiting NK cell activation *via* siglec-mediated signal transduction (76); recruiting myeloid-derived suppressor cells and inflammatory monocytes to the early metastatic niche (54); and mediating the transduction of growth signals presented by immune cells to support metastatic growth (38).

In this study, we demonstrate the possibility of attenuating metastatic ability of two mouse mammary tumor cell lines, 4T1 (BALB/c background) and 6DT1 (FVB background) by targeting *Cmas*. Based on patient survival data, we are also optimistic about the potential of targeting *CMAS* in human cancer, especially since another study has already indicated that tumor growth is impacted by *CMAS* knockdown in human breast cancer cells (37). However, the exact mechanism by which metastasis was attenuated, including the aspect(s) of metastasis affected by *Cmas*-KO, remains to be elucidated and will be the focus of a subsequent study.

This study further demonstrates the power of metabolomics in the study of metastasis and highlights the importance of investigating metabolic changes to identify targets for treating metastasis. We expect that future studies on different models of metastasis will yield additional insights into the metabolic features of metastatic tumors, as well as inform the development of strategies to combat metastasis.

## Ethics Statement

This study was carried out in accordance with the recommendations of the Institutional Animal Care and Use Committee (IACUC) of Michigan State University. The protocol was approved by the IACUC.

## Author Contributions

ST performed metabolomics experiments, cell culture, gene knockout experiments, histology image analysis, and data analysis. MO maintained the FVB and BALB/c mice used in the experiments and performed orthotopic cell injections and mouse necropsies. CR performed PyMT mouse cross-breeding and harvested PyMT tumors. KH and SL conceived and coordinated the study. SL designed and supervised the study. All authors contributed to writing, reviewing, and/or revising the manuscript.

## Conflict of Interest Statement

The authors declare that the research was conducted in the absence of any commercial or financial relationships that could be construed as a potential conflict of interest.

## Footnotes

^1^http://www.benchling.com/ (Accessed: Febuary 6, 2018).

^2^http://www.acgtinc.com/ (Accessed: Febuary 6, 2018).

^3^http://tide.nki.nl/ (Accessed: Febuary 6, 2018).

^4^http://www.kmplot.com.

^5^http://fiji.sc/.

## Supplementary Material

Data Sheet 1 Metabolomics data for PyMT-driven tumors generated in different mouse maternal backgrounds. A summary of the sample groups is provided in the “sampleIDs” sheet. The “fullmethod_AreaTop” and “CBZ_AreaTop” sheets contain initial peak-picking output from non-derivatized full-method and CBZ-derivatized datasets, respectively. The “average-scaled_combine” sheet contains the result of separately average-normalizing the two datasets above and then combining them for analysis. The “barplots” and “ttest & summary” sheets display results from statistical data analysis.

Data Sheet 2 Quantification data for UDP-N-acetylglucosamine, sialic acid, and CMP-sialic acid in 4T1, 4T07, and 67NR cell lines.

Data Sheet 3 13C-labeling time course data for UDP-N-acetylglucosamine, sialic acid, and CMP-sialic acid in 4T1, 4T07, and 67NR cell lines.

Image 1 Isotopic abundances of species incorporating ^13^C-glucose labeled hexose ring. Highly metastatic 4T1 cells, slightly metastatic 4T07 cells, and non-metastatic 67NR cells were incubated with ^13^C-glucose media and extracted for metabolites at indicated time points. Labeled abundances were calculated by first summing the isotopic ratios of species showing ^13^C-glucose labeled hexose rings (reflecting metabolic flux through the hexosamine-sialic acid pathway), then multiplying the ratio sum by the metabolite’s unlabeled intensity at time zero. Abundances of **(A)** uridine diphosphate (UDP)-N-acetylglucosamine, **(B)** sialic acid, and **(C)** cytidine monophosphate (CMP)-sialic acid are represented relative to 4T07 averages for clarity. Values are the average of three biological replicates at each time point. Error bars represent SD.

Image 2 Sequencing confirmation of *Cmas* knockout clones. A 773bp region enclosing the CRISPR target site was sequenced and analyzed with Tracking of Indels by Decomposition (TIDE) (see text footnote 3). TIDE generates indel spectra showing the predicted indels in a mixed population of sequences, as well as the proportion of each sequence. The *R*^2^ value indicates the total proportion of sequences that were successfully analyzed. *p*-values are calculated for each indel to indicate confidence in the existence of that indel, and indels with high confidence (*p*-value < 0.001) are highlighted in red.

Image 3 Western blotting confirmation of *Cmas* knockout clones. **(A)** 4T1 cell line clones. **(B)** 6DT1 cell line clones. Each lane was loaded with the same amount of protein (30 µg). To detect Cmas protein expression, rabbit polyclonal anti-Cmas HPA039905 (Sigma-Aldrich) and horseradish peroxidase (HRP)-linked goat anti-rabbit IgG (heavy and light chain) antibody (Cell Signaling) were used for primary and secondary staining, respectively. Vinculin was used as a loading control (stained with anti-vinculin E1E9V; Cell Signaling). Clones selected for orthotopic injection are marked with middle dots (•).

Image 4 Stripchart showing individual lung met counts for *Cmas*-WT and *Cmas*-KO. Each data point is the total number of discrete metastatic lesions counted over six sections, taken at 200 µm depth intervals, of the same lung. The *Cmas*-KO sample marked with a red arrow was determined to be an outlier according to chi-squared test (*p*-value = 0.01125) and Grubbs’ test for outliers (*p*-value = 0.0003802).

Image 5 RNA-seq data for PyMT tumors of differing metastatic propensities generated under different mouse backgrounds. Gene expression represented by normalized sequence reads for eight hexosamine/sialic acid pathway genes (Gfpt1, Gfpt2, Pgm3, Uap1, Gne, Nans, Nanp, and Cmas) are shown. Data points are grouped according to metastatic propensity of the tumors (high metastatic: BL10, CAST; low metastatic: BL6, MOLF).

Image 6 Microarray gene expression profiling data from GSE11259 dataset. Probe intensities of nine hexosamine/sialic acid pathway genes (Gfpt1, Gfpt2, Pgm3, Uap1, Gne, Nans, Nanp, Cmas, and Slc35a1) were compared between highly metastatic 4T1 and non-metastatic 67NR tumors generated by cell line implantation into BALB/c mice. Microarray CEL data files were analyzed using Transcriptome Analysis Console (TAC) software by ThermoFisher Scientific.

Image 7 Gene expression profiling data from GSE42272 microarray dataset. Probe intensities of nine hexosamine/sialic acid pathway genes (Gfpt1, Gfpt2, Pgm3, Uap1, Gne, Nans, Nanp, Cmas, and Slc35a1) were compared between highly metastatic 4T1 and non-metastatic 67NR tumors generated by cell line implantation into BALB/c mice. Microarray CEL data files were analyzed using Transcriptome Analysis Console (TAC) software by ThermoFisher Scientific.

Image 8 Gene expression profiling data from GSE19086 microarray dataset. Probe intensities of eight hexosamine/sialic acid pathway genes (Gfpt1, Gfpt2, Pgm3, Uap1, Gne, Nans, Cmas, and Slc35a1) were compared between highly metastatic 4T1, low-metastatic 4T07 and non-metastatic 67NR tumors generated by cell line implantation into BALB/c mice. Microarray CEL data files were analyzed using Transcriptome Analysis Console (TAC) software by ThermoFisher Scientific.

Image 9 Western blot for Cmas in syngeneic Balb/c tumor-derived cell lines. Low-metastatic 67NR cells showed low Cmas protein expression in agreement with RNA-seq gene expression data. Note that the second technical replicate of 4T07 showed low signal for both vinculin and Cmas bands, likely due to an incomplete transfer from electrophoretic gel to the blotting membrane.
