# Supplementary figures and images for: Sialic Acid Metabolism: A Key Player in Breast Cancer Metastasis Revealed by Metabolomics
Source: Front Oncol. 2018 May 28;8:174. doi: 10.3389/fonc.2018.00174 (PMC5985449; doi:10.3389/fonc.2018.00174)

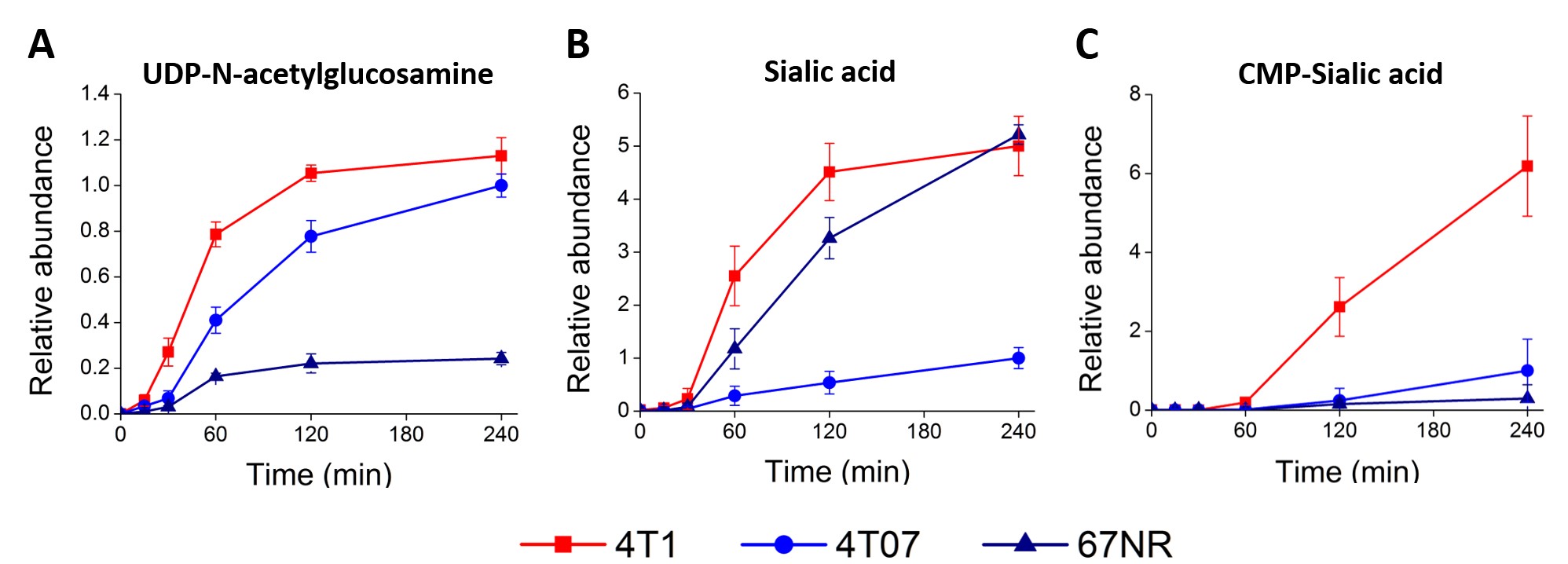

Supplement: Image 1 — Isotopic abundances of species incorporating 13C-glucose labeled hexose ring. Highly metastatic 4T1 cells, slightly metastatic 4T07 cells, and non-metastatic 67NR cells were incubated with 13C-glucose media and extracted for metabolites at indicated time points. Labeled abundances were calculated by first summing the isotopic ratios of species showing 13C-glucose labeled hexose rings (reflecting metabolic flux through the hexosamine-sialic acid pathway), then multiplying the ratio sum by the metabolite’s unlabeled intensity at time zero. Abundances of (A) uridine diphosphate (UDP)-N-acetylglucosamine, (B) sialic acid, and (C) cytidine monophosphate (CMP)-sialic acid are represented relative to 4T07 averages for clarity. Values are the average of three biological replicates at each time point. Error bars represent SD. [file image_1.jpeg]

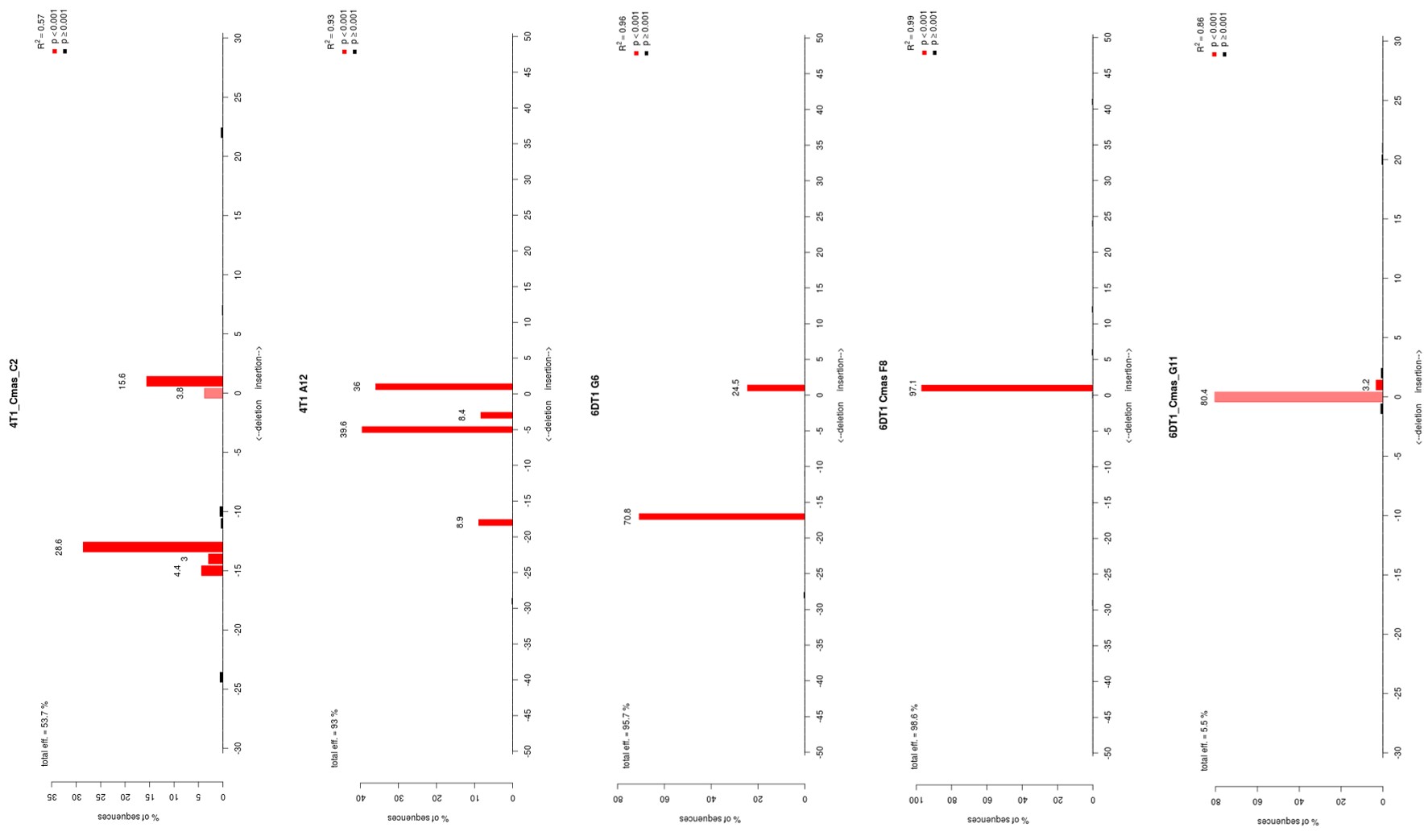

Supplement: Image 2 — Sequencing confirmation of Cmas knockout clones. A 773bp region enclosing the CRISPR target site was sequenced and analyzed with Tracking of Indels by Decomposition (TIDE) (see text footnote 3). TIDE generates indel spectra showing the predicted indels in a mixed population of sequences, as well as the proportion of each sequence. The R2 value indicates the total proportion of sequences that were successfully analyzed. p-values are calculated for each indel to indicate confidence in the existence of that indel, and indels with high confidence (p-value < 0.001) are highlighted in red. [file image_2.jpeg]

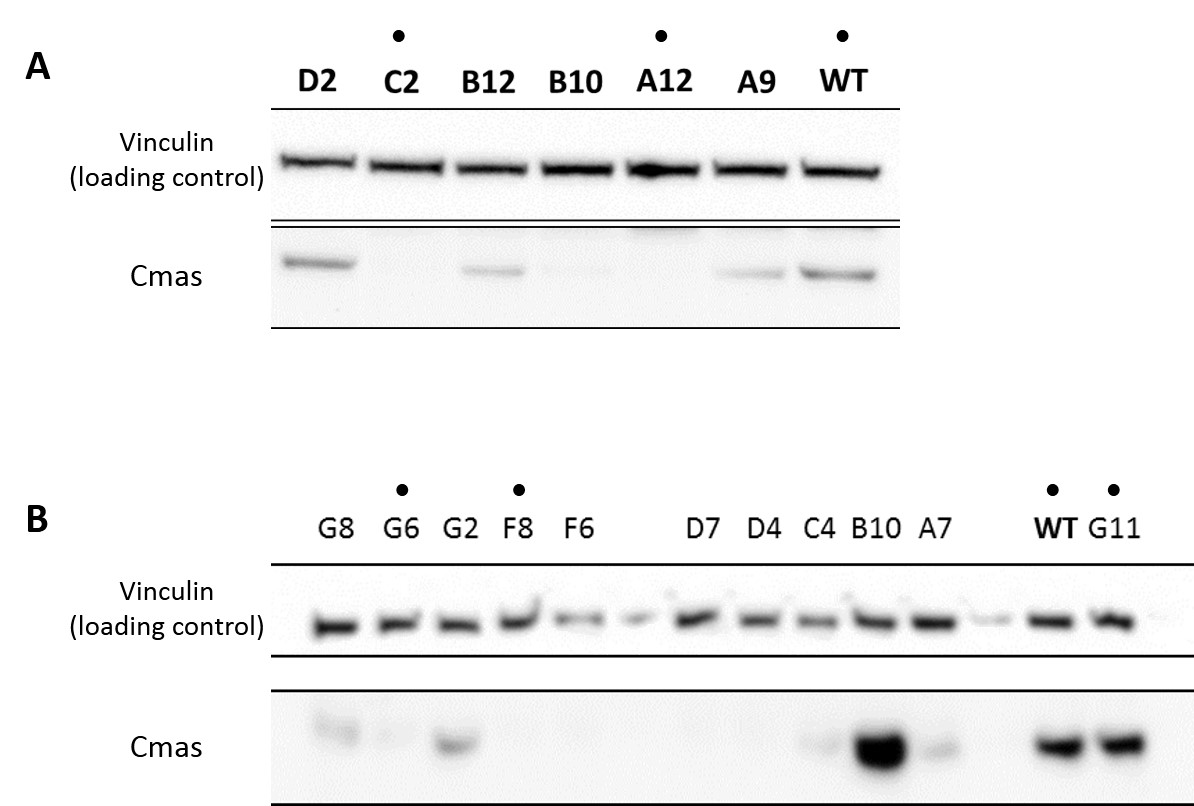

Supplement: Image 3 — Western blotting confirmation of Cmas knockout clones. (A) 4T1 cell line clones. (B) 6DT1 cell line clones. Each lane was loaded with the same amount of protein (30 µg). To detect Cmas protein expression, rabbit polyclonal anti-Cmas HPA039905 (Sigma-Aldrich) and horseradish peroxidase (HRP)-linked goat anti-rabbit IgG (heavy and light chain) antibody (Cell Signaling) were used for primary and secondary staining, respectively. Vinculin was used as a loading control (stained with anti-vinculin E1E9V; Cell Signaling). Clones selected for orthotopic injection are marked with middle dots (•). [file image_3.jpeg]

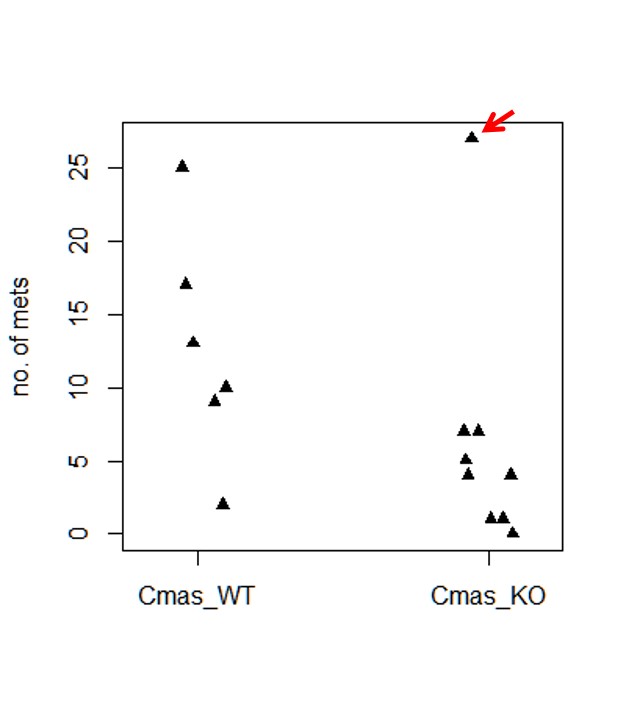

Supplement: Image 4 — Stripchart showing individual lung met counts for Cmas-WT and Cmas-KO. Each data point is the total number of discrete metastatic lesions counted over six sections, taken at 200 µm depth intervals, of the same lung. The Cmas-KO sample marked with a red arrow was determined to be an outlier according to chi-squared test (p-value = 0.01125) and Grubbs’ test for outliers (p-value = 0.0003802). [file image_4.jpeg]

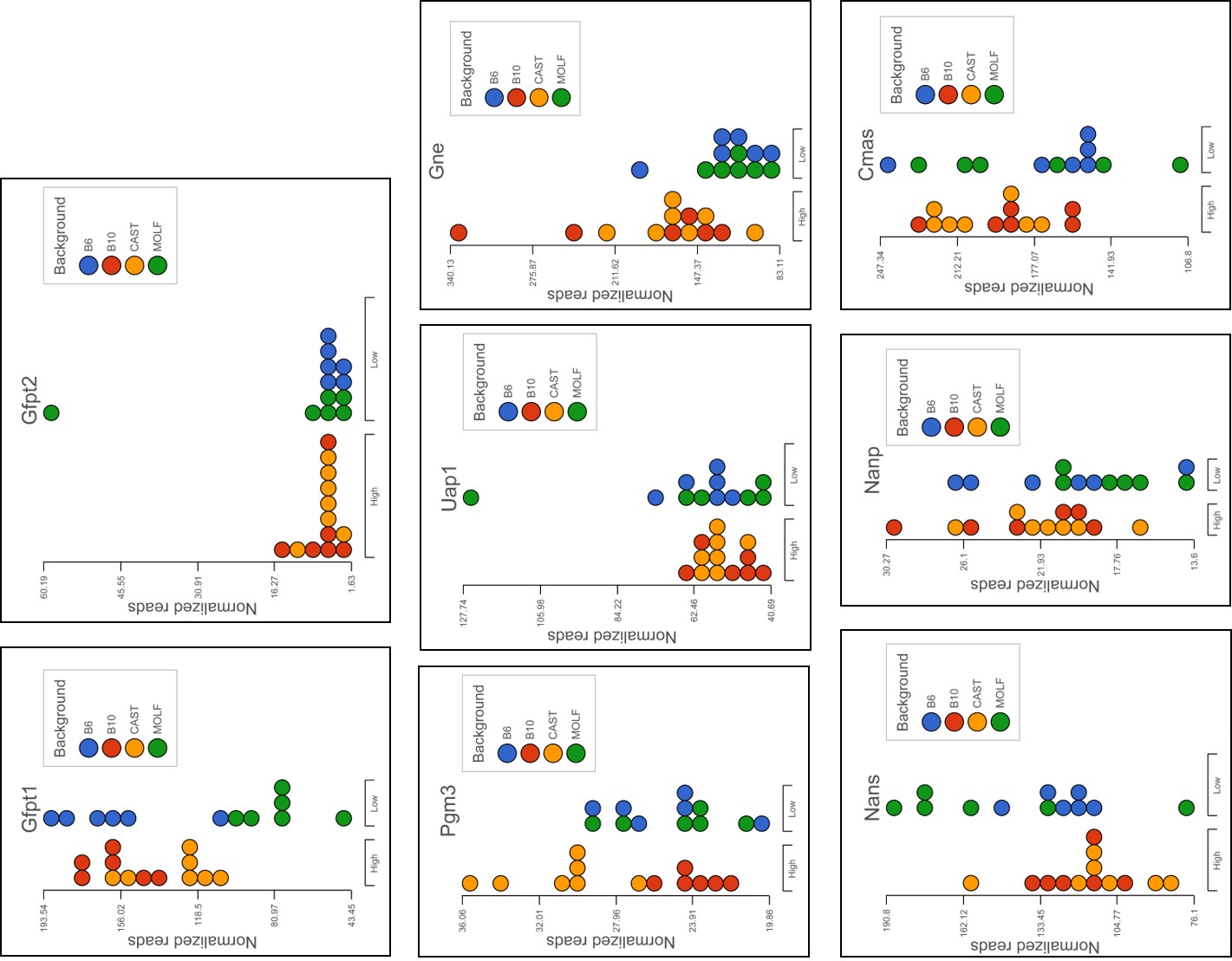

Supplement: Image 5 — RNA-seq data for PyMT tumors of differing metastatic propensities generated under different mouse backgrounds. Gene expression represented by normalized sequence reads for eight hexosamine/sialic acid pathway genes (Gfpt1, Gfpt2, Pgm3, Uap1, Gne, Nans, Nanp, and Cmas) are shown. Data points are grouped according to metastatic propensity of the tumors (high metastatic: BL10, CAST; low metastatic: BL6, MOLF). [file image_5.jpeg]

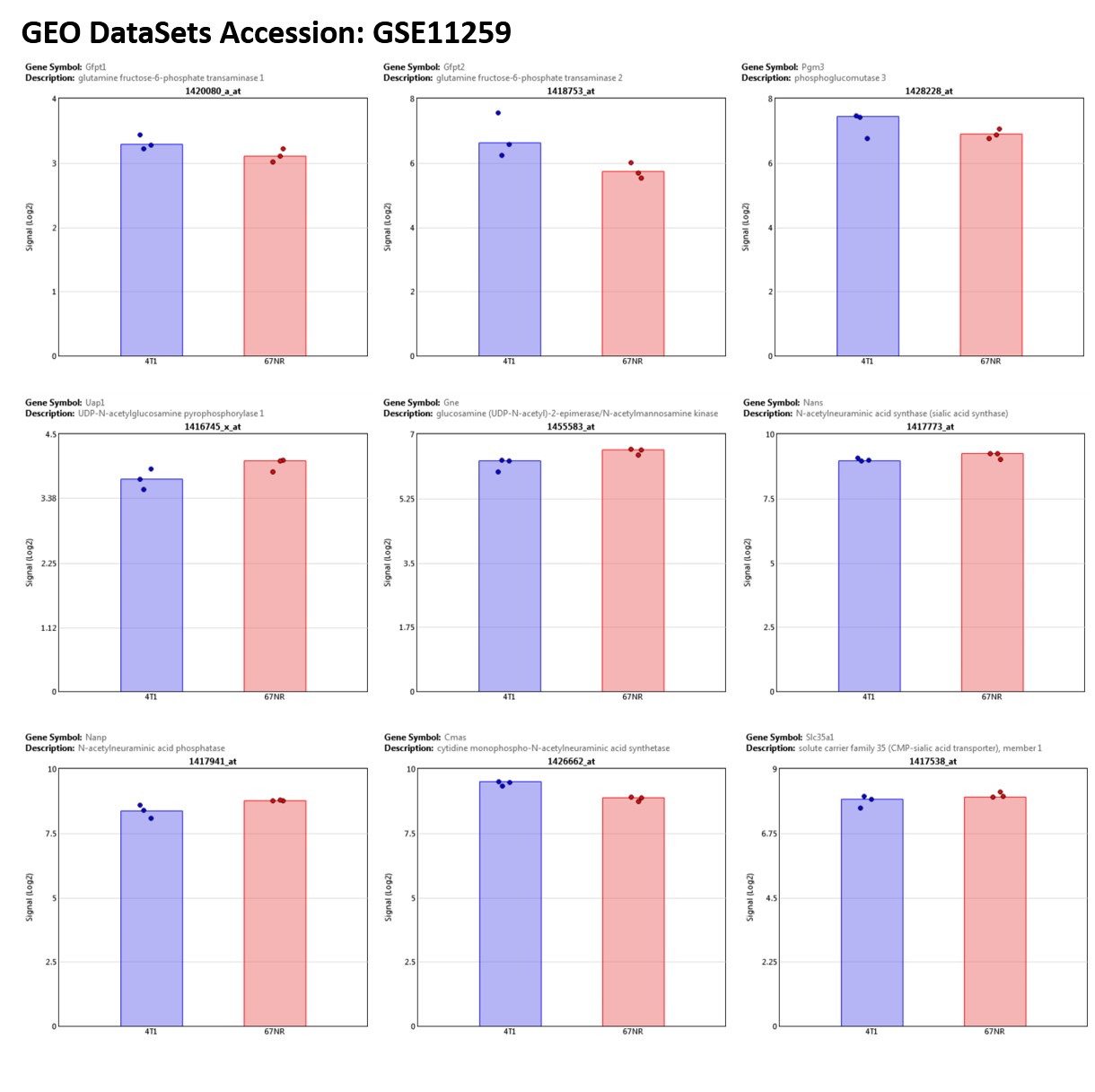

Supplement: Image 6 — Microarray gene expression profiling data from GSE11259 dataset. Probe intensities of nine hexosamine/sialic acid pathway genes (Gfpt1, Gfpt2, Pgm3, Uap1, Gne, Nans, Nanp, Cmas, and Slc35a1) were compared between highly metastatic 4T1 and non-metastatic 67NR tumors generated by cell line implantation into BALB/c mice. Microarray CEL data files were analyzed using Transcriptome Analysis Console (TAC) software by ThermoFisher Scientific. [file image_6.jpeg]

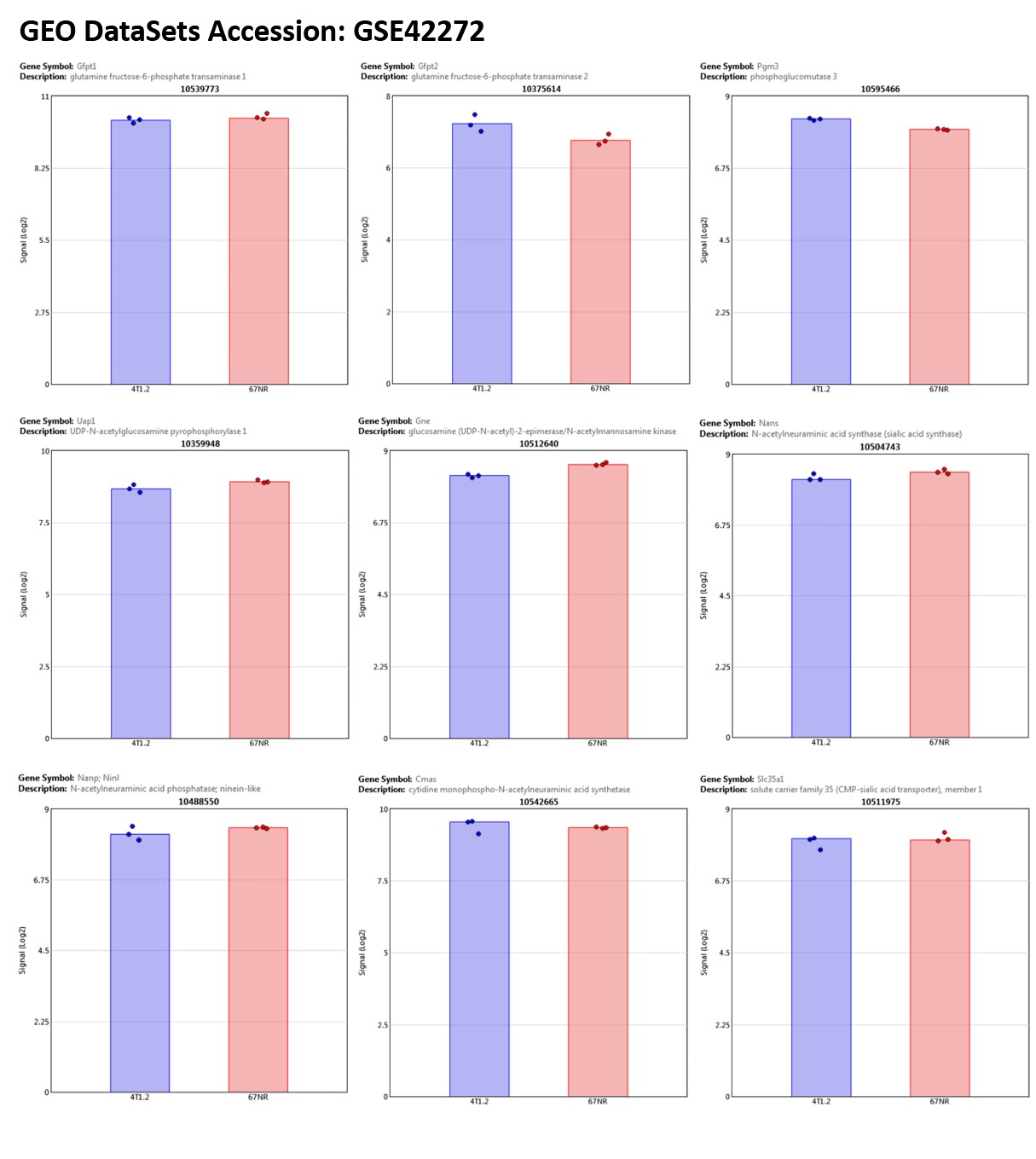

Supplement: Image 7 — Gene expression profiling data from GSE42272 microarray dataset. Probe intensities of nine hexosamine/sialic acid pathway genes (Gfpt1, Gfpt2, Pgm3, Uap1, Gne, Nans, Nanp, Cmas, and Slc35a1) were compared between highly metastatic 4T1 and non-metastatic 67NR tumors generated by cell line implantation into BALB/c mice. Microarray CEL data files were analyzed using Transcriptome Analysis Console (TAC) software by ThermoFisher Scientific. [file image_7.jpeg]

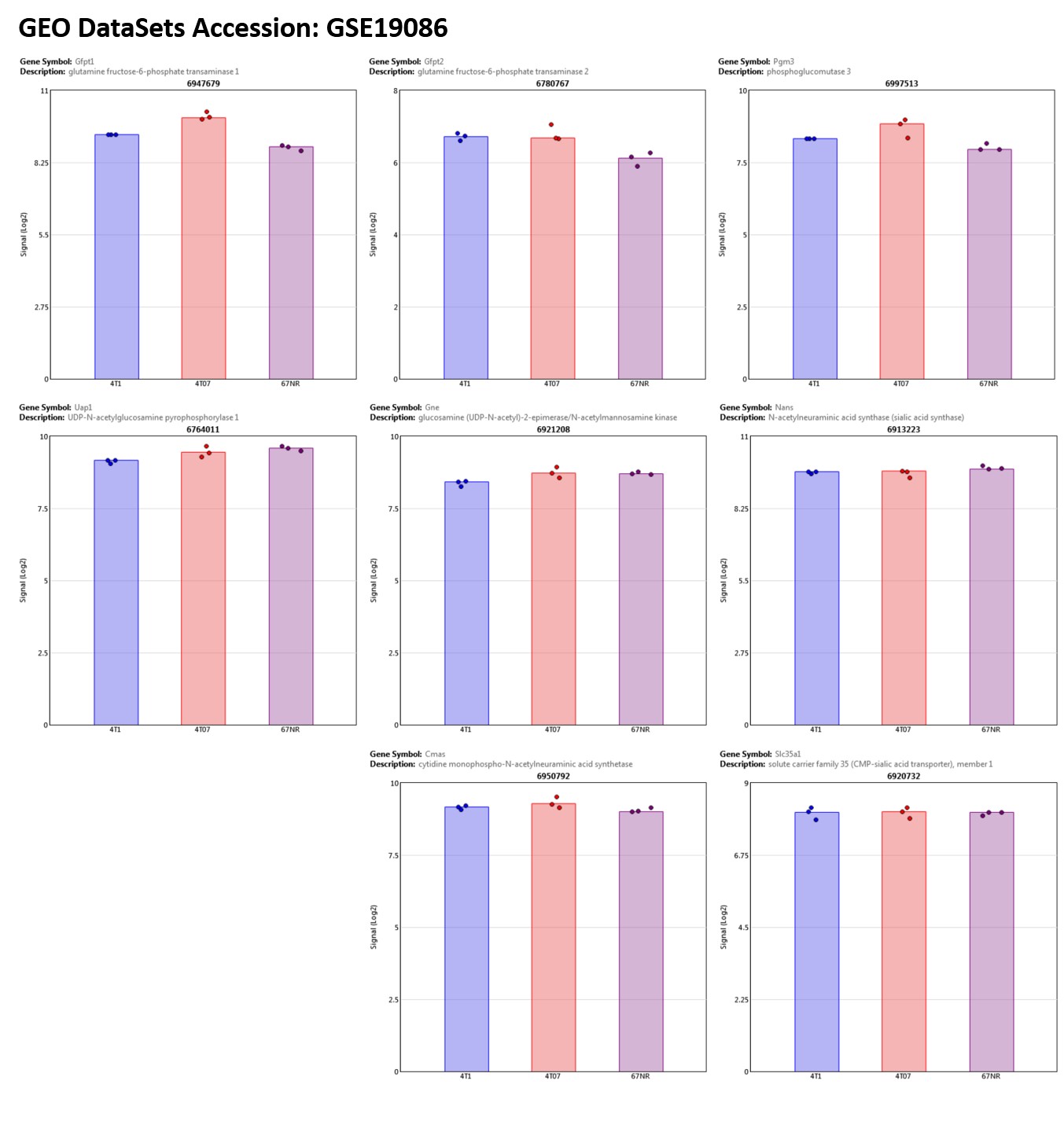

Supplement: Image 8 — Gene expression profiling data from GSE19086 microarray dataset. Probe intensities of eight hexosamine/sialic acid pathway genes (Gfpt1, Gfpt2, Pgm3, Uap1, Gne, Nans, Cmas, and Slc35a1) were compared between highly metastatic 4T1, low-metastatic 4T07 and non-metastatic 67NR tumors generated by cell line implantation into BALB/c mice. Microarray CEL data files were analyzed using Transcriptome Analysis Console (TAC) software by ThermoFisher Scientific. [file image_8.jpeg]

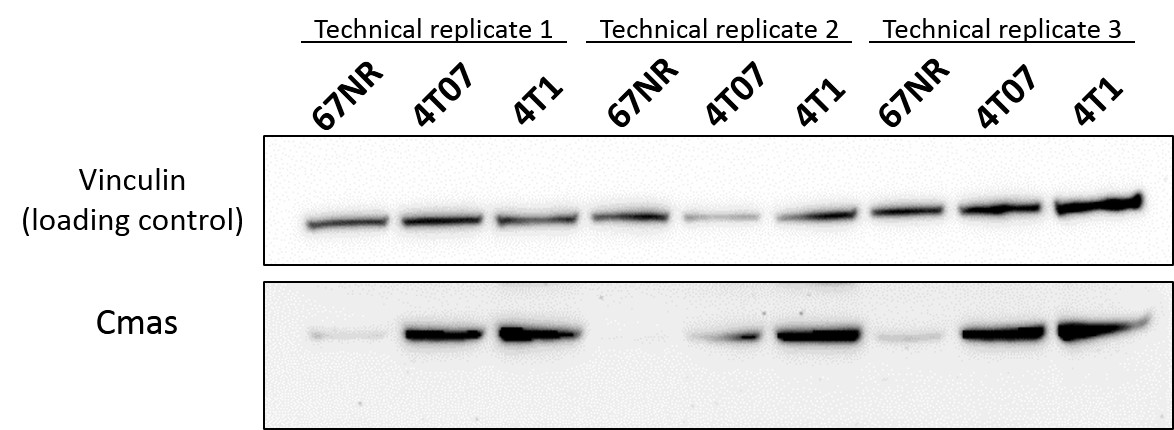

Supplement: Image 9 — Western blot for Cmas in syngeneic Balb/c tumor-derived cell lines. Low-metastatic 67NR cells showed low Cmas protein expression in agreement with RNA-seq gene expression data. Note that the second technical replicate of 4T07 showed low signal for both vinculin and Cmas bands, likely due to an incomplete transfer from electrophoretic gel to the blotting membrane. [file image_9.jpeg]
